# C_6_-ceramide nanoliposome suppresses tumor metastasis by eliciting PI3K and PKCζ tumor-suppressive activities and regulating integrin affinity modulation

**DOI:** 10.1038/srep09275

**Published:** 2015-03-20

**Authors:** Pu Zhang, Changliang Fu, Yijuan Hu, Cheng Dong, Yang Song, Erqun Song

**Affiliations:** 1 College of Pharmaceutical Sciences, Southwest University, Chongqing, China, 400715; 2 Department of Bioengineering, Pennsylvania State University, University Park, PA, US 16801; 3 Rollins School of Public Health, Emory University, Atlanta, GA, US 30322

## Abstract

Nanoliposomal formulation of C_6_-ceramide, a proapoptotic sphingolipid metabolite, presents an effective way to treat malignant tumor. Here, we provide evidence that acute treatment (30 min) of melanoma and breast cancer cells with nanoliposomal C_6_-ceramide (NaL-C_6_) may suppress cell migration without inducing cell death. By employing a novel flow migration assay, we demonstrated that NaL-C_6_ decreased tumor extravasation under shear conditions. Compared with ghost nanoliposome, NaL-C_6_ triggered phosphorylation of PI3K and PKCζ and dephosphorylation of PKCα. Concomitantly, activated PKCζ translocated into cell membrane. siRNA knockdown or pharmacological inhibition of PKCζ or PI3K rescued NaL-C_6_-mediated suppression of tumor migration. By inducing dephosphorylation of paxillin, PKCζ was responsible for NaL-C_6_-mediated stress fiber depolymerization and focal adhesion disassembly in the metastatic tumor cells. PKCζ and PI3K regulated cell shear-resistant adhesion in a way that required integrin α_v_β_3_ affinity modulation. In conclusion, we identified a novel role of acute nanoliposomal ceramide treatment in reducing integrin affinity and inhibiting melanoma metastasis by conferring PI3K and PKCζ tumor-suppressive activities.

Ceramide is a sphingolipid-derived second messenger in cell membrane in response to inflammation and stress^1^. It is an integral part of cellular differentiation, proliferation and apoptosis pathways. Studies indicated that endogenous ceramide metabolisms were downregulated in several cancers. Unlike long-chain ceramides, short-chain ceramides could induce cell death, which is useful for therapeutic applications in cancer. Nanoliposomal formulation improved bioavailability and solubilization of hexanoyl-D-*erythro*-sphingosine(C_6_-ceramide)^2^. Exogenous delivery of C_6_ resulted in accumulation of ceramide in structured membrane microdomains which contained caveolin-1-enriched lipid rafts^3^. High doses (>20 μM) of nanoliposomal C_6_-ceramide (NaL- C_6_) with long-term incubation (8 ~ 24 hr) inhibited *in vitro* and *in vivo* growth of breast cancer, pancreatic cancer, chronic lymphocytic leukemia, hepatocellular carcinoma and melanoma^2^,^4^,^5^,^6^,^7^. Of note, nanoliposome-formulated ceramide significantly decreased breast carcinoma, MDA-MB-231 cell proliferation as compared with nonliposomal ceramide^2^. However, within circulation, tumor extravasation occurs very rapidly, especially in face of hydrodynamic force^8^,^9^. It is unknown whether nanoliposomal C_6_-ceramide play roles in inhibiting tumor migration and metastasis upon this short encounter with tumor cells in blood stream.

The protein kinase C (PKC) family consists of at least 11 members being classified into three groups: classical, novel, and atypical PKCs, depending on their requirement for regulation by calcium and diacylglycerol (DAG)^10^. PKCζ, highly expressed in breast cancer cells, belongs to the atypical group, and is independent of calcium and DAG for its activities. Upon activation, PKCζ can translocate from cytosol to cell membrane^11^. PKCζ is directly or indirectly regulated by several lipids including ceramides and phosphatidylinositol 3,4,5-triphosphate (PIP3)^12^. Studies suggested that breast carcinoma cell invasiveness and metastasis were dependent on PKCζ activation^13^.

Adhesion to and extravasation through the endothelial lining of blood vessels are prerequisite for establishment of tumor metastasis. Under hydrodynamic conditions, tumor cells undergo multistep adhesive interactions with vascular endothelium. This involves sialylated molecule-mediated initial tethering and integrin-mediated firm adhesion of tumor cells^9^,^14^,^15^. Nevertheless, some tumor cell lines, like melanoma, does not express selectin-ligand sialyl-Lews^a/x^ at sufficient levels to mediate tethering and rolling of tumor cells^16^. Therefore, they hijack polymorphonuclear neutrophils (PMNs) or fibrin to bridge them into close proximity to the endothelial cells, thereby facilitating their subsequent migration through endothelial cells^17^,^18^,^19^,^20^,^21^. Like melanoma cells, metastatic breast cancer cell, MDA-MB-231 is negative for sialofucosylated selectin ligands and integrin β_1_ and β_2_ integrins, like lymphocyte function-associated antigen-1 (LFA-1), Mac-1 and very late antigen-4 (VLA-4). Therefore, they were deficient in binding to endothelial intercellular adhesion molecule-1 (ICAM-1) and vascular cell adhesion molecule-1 (VCAM-1). Integrin α_v_β_3_ was found to be expressed on MDA-MB-231 by other groups^22^,^23^ and ourselves. Integrin α_v_β_3_ plays important roles in breast cancer metastasis^24^,^25^. Integrin α_v_β_3_ can form bonds with fibrinogen which served as connecting ligands facilitating melanoma and breast cancer adhesion to endothelium in flow^18^,^25^,^26^.

To evaluate the effect of short-term nanoliposomal ceramide treatment on breast cancer and melanoma migration, we studied MDA-MB-231 and Lu1205 cell static and flow migration potencies in response to 30 min NaL-C_6_ treatment. NaL-C_6_ attenuated tumor migration in a dose-dependent manner. By using mutant constructs, pharmacological inhibitors and short interference RNA (siRNA) knockdown, we discovered that NaL-C_6_-mediated PKCζ and PI3K phosphorylation and PKCα dephosphorylation were responsible for reduced cell migration. As the activation of PKC isoforms and PI3K were conventionally conceived of augmenting malignancy of tumors, we uncovered a novel role of PKCζ and PI3K as tumor suppressors. The strategies of activating PKCζ might potentiate the therapeutic effect of nanoliposomal ceramide to treat tumor metastasis.

## Results

### Acute treatment with C_6_ nanoliposome suppressed tumor migration

**Figure 1:**
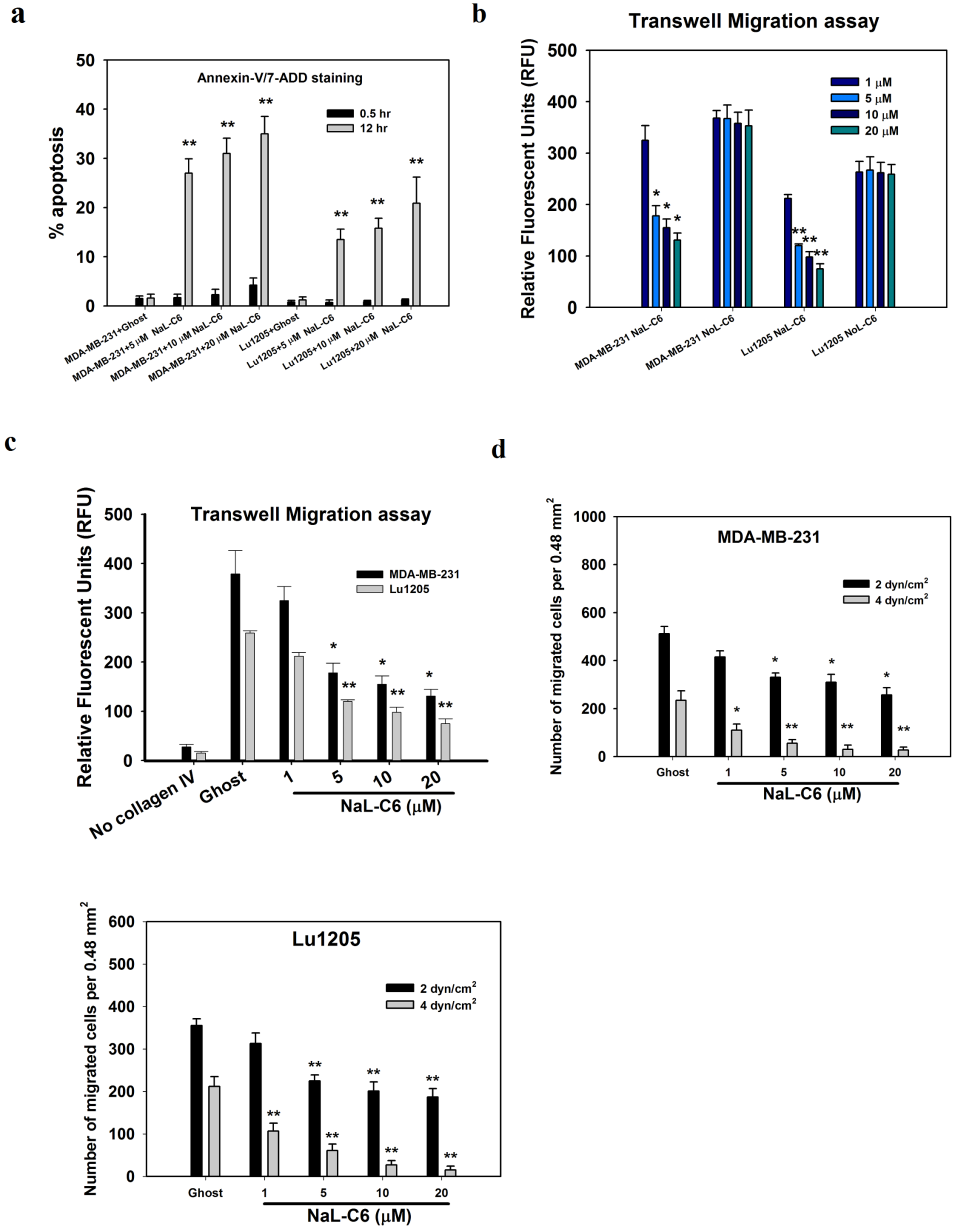
C_6_-ceramide nanoliposomes suppressed MDA-MB-231 and Lu1205 migration in a dose-dependent manner. (a) MDA-MB-231 or Lu1205 cells did not undergo apoptosis after receiving acute treatment (30 min) of 5 μM, 10 μM or 20 μM ceramide nanoliposome.12-hr treatment with ceramide nanoliposomes induced tumor cell death. % of cells undergoing apoptosis was analyzed with Annexin-v/7-ADD staining and flow cytometry. Values were mean ± SEM. n = three replicates. **p < 0.01 compared with ghost control. (b–c) Liposomal C_6_ delivery augmented the anti-migration activity of C_6_-ceramide. MDA-MB-231 or Lu1205 cells received 1 μM, 5 μM, 10 μM or 20 μM liposomal (NaL-C_6_) or nonliposomal C_6_-ceramide (NoL-C_6_) (b) or ghost nanoliposome (c) for 30 min before being used for transwell migration assays. The amounts of migrated cells were determined by calcein AM staining after 4-hr onset of experiment and were expressed as RFU. 100 mg/ml collagen IV was used as chemoattractant in the bottom well. In no collagen IV group, DMEM + 0.1% BSA was added into chemoattractant wells instead. Results were expressed as mean ± SEM. n = three replicates.*p < 0.05, **p < 0.01 compared with control for each cell type.(d) NaL-C_6_ attenuated MDA-MB-231 or Lu1205 cell transendothelial migration in a dose-dependent manner under flow conditions. Tumor cells were incubated with 1 μM, 5 μM, 10 μM or 20 μM NaL-C_6_ or ghost nanoliposome for 30 min before being introduced into flow migration chamber together with 1.5 mg/ml fibrinogen. The flow migration assay was carried out for 4 hr at shear stress of 2 or 4 dyn/cm^2^. The migrated cancer cells were stained and counted at the bottom of filter. Results were expressed as mean ± SEM. n = three replicates.*p < 0.05, **p < 0.01compared with ghost at the same shear stress.

In previous studies, it was demonstrated that C_6_ nanoliposomes at high dosage range and long exposure duration mediated cancer apoptosis and growth arrests^2^,^4^,^5^,^6^,^7^. But it remains elusive whether acute treatment of cancer cells with C_6_ nanoliposomes at low dosage range had any impacts on cell phenotypes. We measured MDA-MB-231 and Lu1205 apoptosis after being treated with a variety of doses of NaL-C_6_ for 30 min and 12 hr^3^,^27^. Upon 30 min 20 μM NaL-C_6_ incubation, only 4% MDA-MB-231 and 1% Lu1205 cells underwent apoptosis (Fig. 1a). In contrast, 20 μM NaL-C_6_ with a long 12 hr of exposure resulted in 35% MDA-MB-231 and 20% Lu1205 apoptosis.

Next, we assessed 4-hr transwell migration of MDA-MB-231 and Lu1205 cells which received nonliposomal (NoL- C_6_) or liposomal C_6_ treatment for 30 min. Liposomal C_6_ were more effective to suppress MDA-MB-231 and Lu1205 cell migration than freely administrated C_6_ (Fig. 1b). This disparity of nonliposomal and liposomal C_6_ effects may be caused by the difference in the resultant accumulation of C_6_ within 30 min timescale^2^. Collagen IV has been reported to function as chemoattractant for melanoma migration and enhance breast cancer motility^28^. In the absence of collagen IV, few MDA-MB-231 or Lu1205 cells migrated to the opposite side of the membrane (28 ± 5 RFU and 21 ± 3 RFU), while 100 mg/ml collagen IV in the bottom well strikingly augmented ghost nanoliposome-treated MDA-MB-231 or Lu1205 cell migration (379 ± 47 RFU and 278 ± 8 RFU) (Fig. 1c). NaL-C_6_ suppressed MDA-MB-231 cell migration in a dose-dependent manner. 5 ~ 20 μM NaL-C_6_ significantly attenuated MDA-MB-231 or Lu1205 transmigration as compared with ghost nanoliposome (*p* < 0.05).

Flow-regulated cancer migration plays important roles in tumor metastasis^14^. To evaluate the effect of nanoliposomal ceramide treatment on cancer migration under hydrodynamic conditions, we utilized a flow migration device which consists of a modified 48-well Boyden chamber and a flow loop^19^,^21^,^29^. To facilitate successful tumor extravasation in flow, a stable adhesion mediated by integrin is required. However, a screening of surface expressions of adhesive molecules revealed that neither MDA-MB-231 nor Lu1205 expressed integrins LFA-1 and Mac-1, the counter-receptors for endothelial ICAM-1 (Table 1)^14^,^30^,^31^.These two cell lines express integrin α_v_β_3_ which is a receptor for plasma protein, fibrinogen. Previous studies suggested that fibrinogen serving as a linker for cell-cell adhesion supported integrin α_v_β_3_-dependent adhesion of melanoma cells to endothelium under flow conditions^18^,^25^,^26^. Therefore, in the current flow migration settings, the transendothelial migratory properties of cancer cells were analyzed in the presence of fibrinogen at physiological concentration of 1.5 mg/ml. As expected, fibrinogen promoted the transmigration of substantial amounts of MDA-MB-231 cells at shear stresses of 2 and 4 dyn/cm^2^ (512 ± 30 and 235 ± 39 cells/0.48 mm^2^ of filter) (Fig. 1d). At both shear stresses, NaL-C_6_ dramatically attenuated MDA-MD-231 and Lu1205 migration compared with ghost nanoliposome in a dose-dependent manner (*p* < 0.05) (Fig. 1d). At high shear stress (4 dyn/cm^2^), 20 μM NaL-C_6_ for 30 min exposure resulted in 8.7 and 14.1 fold reduction of MDA-MB-231 and Lu1205 migration. The data indicated that acute treatment with NaL-C_6_ (30 min) suppressed shear-dependent tumor migration.

### Nanoliposomal C_6_ triggered phosphorylation of PKCζ and PI3K and translocation of PKCζ into cell membrane

**Figure 2:**
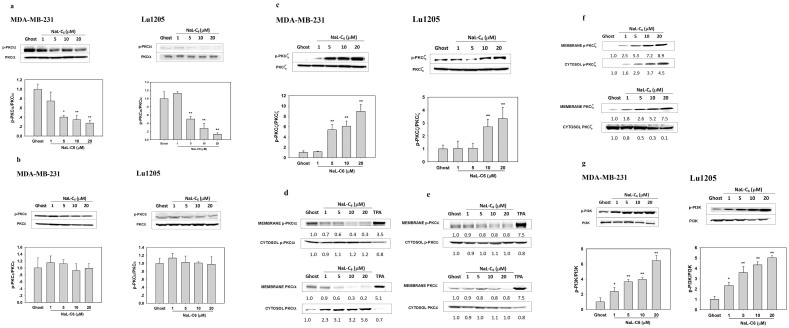
Nanoliposomal C_6_-ceramide induced phosphorylation of PKCζ nd PI3K as well as dephosphorylation of PKCα in a dose-dependent manner. (a–c) PKCζ was phosphorylated in response to acute NaL-C_6_-ceramide treatment. 1 μM, 5 μM, 10 μM or 20 μM NaL-C_6_ or ghost nanoliposomes were incubated with 1 × 10^6^ MDA-MB-231 or Lu1205 cells for 30 min. Then, the cells were subject to western blotting analysis of (a) PKCα, (b)PKCε, and (b)PKCζ phosphorylation levels. Total PKCα, PKCε, and PKCζ were used as loading controls. Data represent three replicates. Densitometric analysis of phosphorylated PKC isoforms with respect to total PKC for each treatment was shown under blots. *p < 0.05, **p < 0.01 compared with ghost control. (d–f) PKCζ was translocated into MDA-MB-231 cell membrane in response to acute NaL-C_6_ treatment. After being incubated with various concentrations of NaL-C_6_ or ghost nanoliposomes, MDA-MB-231 cells were lysed and fractionated to cytosol and membrane components. Phosphorylated and total PKCα, PKCε, or PKCζ in cytosol and membrane fractionations were probed with western blotting. Data represent three replicates. (g) PI3K was phosphorylated in response to acute NaL-C_6_ treatment. MDA-MB-231 or Lu1205 cells were incubated with nanoliposomes in the same way as (a–c). Then, the cells were subject to western blotting analysis of phosphorylated and total PI3K. Data represent three replicates. Densitometric analysis of phosphorylated PI3K with respect to total PI3K for each treatment was shown under blots.*p < 0.05, **p < 0.01 compared with ghost control.

Ceramide is sphingolipid metabolite and accumulates in cell membrane lipid bilayer upon elevated sphingomyelinase activity or *de novo* synthesis^1^,^27^,^32^. To assess the effect of acute NaL-C_6_ treatment on PKC isoform activation in MDA-MB-231 and Lu1205 cells, we determined the phosphorylation states of three PKC isoforms, PKCα, PKCε and PKCζ, which were previously shown to control cancer metastasis^33^,^34^. Phosphorylation of PKCζ threonine 410 residue and PKCα threonine 638 residue in activation loop domain and serine 729 in hydrophobic motifs of PKCε contribute to activation as well as stability of enzymes^10^,^35^. In MDA-MB-231 and Lu1205 cells, PKCα and PKCε were constitutively phosphorylated at residues Thr638 and Ser729, respectively, while PKCζ was marginally phosphorylated at Thr410 (Fig. 2a–c). NaL-C_6_ treatment reduced PKCα phosphorylation but enhanced PKCζ phosphorylation with a maximum effect at 20 μM in both cell lines (Fig. 2a and c). In contrast, PKCε phosphorylation level was not changed by NaL-C_6_ treatment (Fig. 2b).

Previous studies suggested that phosphorylated PKC isoforms translocate to cell membrane to regulate cell behaviors^11^. Therefore, we evaluated the subcellular distribution of three PKC isoforms in response to nanoliposome treatment. Compared with ghost nanoliposome, NaL-C_6_ reduced the amounts of phosphorylated and total PKCα in membrane fraction of tumor cells (Fig. 2d). Total PKCα translocated into cytosolic fraction. In addition, phosphorylated membrane PKCα was decreased with increasing doses of NaL-C_6_ treatment. The distributions of both phosphorylated and total PKCε were not affected by NaL-C_6_ treatment (Fig. 2e). As a positive control, 12-O-tetradecanoylphorbol-13-acetate (TPA) induced a membrane translocation of PKCα and PKCε. On the other hand, acute NaL-C_6_ treatment resulted in translocation of total PKCζ from cytosol to cell membrane (Fig. 2f). Concomitantly, phosphorylation levels of PKCζ was increased in both cytosol and cell membrane. 20 μM NaL-C_6_ treatment resulted in a peak PKCζ phosphorylation in the cell membrane.

Since the phosphorylation of PKC is usually associated with PI3K activation, we next determined the phosphorylation states of PI3K in MDA-MB-231 and Lu1205 cells^35^,^36^. 30 min NaL-C_6_ exposure elevated the phosphorylation levels of PI3K in a dose-dependent manner (Fig. 2g). A peaked level of PI3K phosphorylation was observed as a consequence of 20 μM NaL-C_6_ treatment. Thus, the activity of PI3K paralleled that of PKCζ in response to NaL-C_6_ treatment (Fig. 2a).

### PKCζ was essential for nanoliposomal C_6_-regulated tumor migration

**Figure 3:**
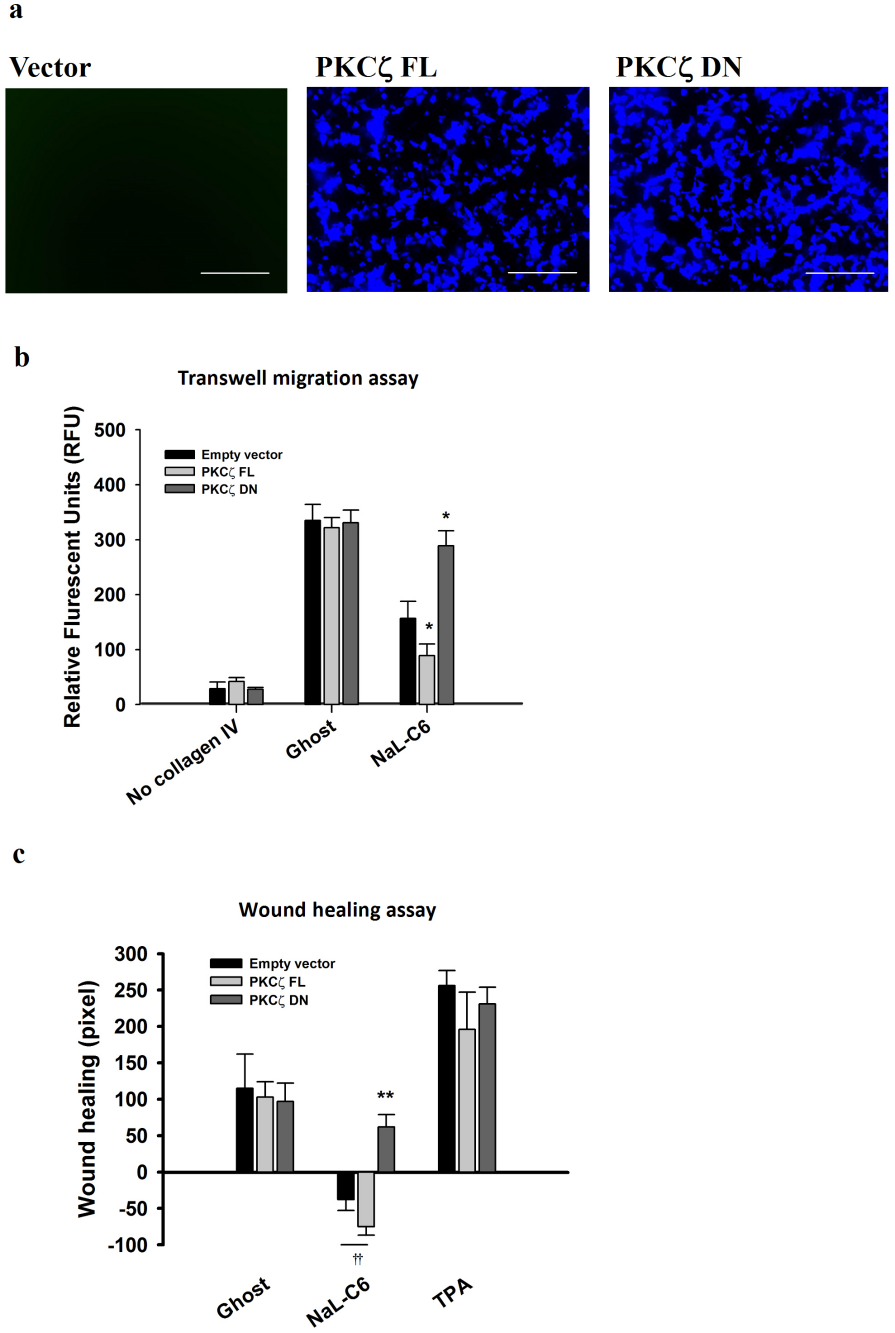
NaL-C_6_-mediated inhibition of MDA-MB-231 migration was dependent on functional activity of PKCζ. (a) Empty vector, PKCζ FL, or PKCζ DN were transfected into MDA-MB-231 cells. The transfection efficiency of these constructs was detected with anti-HA and anti-Alexa 350 staining. >95% cells received target genes. The migration of empty vector, PKCζ FL, or PKCζ DN transfected cells in response to NaL-C_6_ treatment was assessed by transwell migration assay (b) and wound healing assay (c). (b) For transwell migration assays, plasmid-transfected MDA-MB-231 cells were treated with ghost or 20 μM NaL-C_6_ for 30 min before being loaded into transwell inserts. Results were expressed as mean ± SEM. n = three replicates.*p < 0.05 compared with empty vector control. (c)PKCζ DN rescued suppressive effect of NaL-C_6_ on cell wound healing capacity. For wound healing assays, confluent cell monolayers in 6 well plates were scratched and the area (in number of pixels) that cells migrated into the wound over a period of 12 hr was measured. Plasmid-transfected MDA-MB-231 cells were treated with ghost liposome, 20 μM NaL-C_6_, or 200 nM TPA for 30 min before assay. Results were expressed as mean ± SEM. n = three replicates.**p < 0.01,††*p* < 0.01compared with NaL-C_6_+vec.

Since NaL-C_6_ induced PKCζ phosphorylation and accumulation of phosphorylated PKCζ in cell membrane, we hypothesized that PKCζ might play a role in NaL-C_6_-induced suppression of cancer migration. To verify this, we transfected MDA-MB-231 cells with full length PKCζ (PKCζ FL) construct and dominantly negative PKCζ(PKCζ DN) construct which is kinase-defective and contains a point mutation in its kinase domain to assess PKCζ function in cell migration. A 95% transfection efficiency was achieved at the time of functional assays as measured by fluorescently staining HA (constructs were tagged with hemagglutinin (HA)) (Fig. 3a). In the presence of ghost nanoliposomes, PKCζ FL and PKCζ DN overexpression had no effect on cell static transwell migration (Fig. 3b). In sharp contrast, PKCζ FL potentiated suppressive effect of 20 μM nanoliposomal ceramide on MDA-MB-231 transmigration, while PKCζ DN rescued MDA-MB-231 transmigration suppressed by NaL-C_6_ (*p* < 0.05).

Motility is a required process for invasion of tumor cells through the surrounding stroma. To determine whether PKCζ activation was required for regulation of cell motility, wound healing assays were conducted with mutant construct-transfected MDA-MB-231 cells. In consistent with transwell migration assays, PKCζ FL or PKCζ DN overexpression had no effect on wound healing capability of ghost nanoliposome-treated cells (Fig. 3c). 20 μM NaL-C_6_ treatment for 30 min increased the size of initial wounded area. This suggests that NaL-C_6_ treatment resulted in actomyosin-mediated contraction of cells. PKCζ FL overexpression further reduced the wound healing capacities of nanoliposomal ceramide-treated cells (*p* < 0.01). Conversely, PKCζ DN-transfected cells briskly migrated into the wound area, reaching 60% sealing at 12 hr, after wound scratch (Fig. 3c). Upon TPA stimulation, the wound healing rates of cells receiving vector, PKCζ FL and PKCζ DN constructs were comparable.

**Figure 4:**
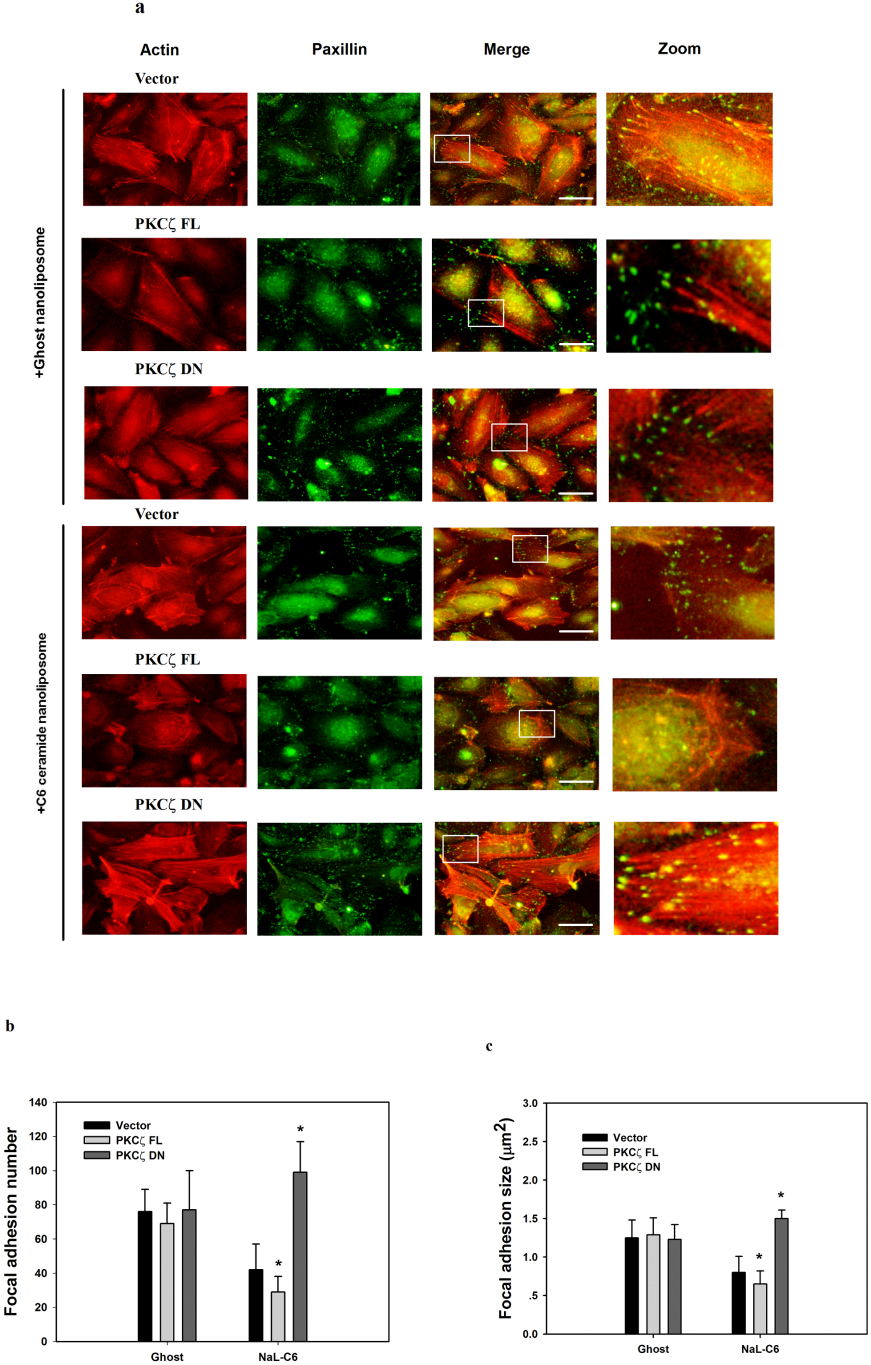
NaL-C_6_ reduced stress fiber formation and inhibited focal adhesion disassembly through PKCζ activation. (a) Vector, PKCζ FL or PKCζ DN-transfected MDA-MB-231 cells were treated with ghost or 20 μM NaL-C_6_ for 30 min. Cells were stained with rhodamine-phalloidin and paxillin antibody. The right panel shows magnified views of the boxed area in the merged images. Bar = 10 μm. (Geen = paxillin, red = F-actin). To verify the presence of PKCζ in given individual cells, the cells were further subject to anti-HA and anti-Alexa 350 staining. The cells shown in the images were all overexpressing target constructs. (b–c) Quantification of the average number and size (μm^2^) of paxillin-containing focal adhesions in vector, PKCζ FL or PKCζ DN-transfected cells treated with 20 μM C_6_-ceramide or ghost nanoliposomes for 30 min using ImageJ software. 12 cells were analyzed per condition in each experiment. Results were expressed as mean ± SEM. *p < 0.05 compared with vector control.

Next, we analyzed the dynamics of actin cytoskeleton and focal adhesion, which are required to maintain cell shapes and promote cell migration. Ghost-treated cells exhibited thick stress fibers which traversed the cell body (Fig. 4a). In addition, in vector, PKCζ FL and PKCζ DN-transfected cells, the appearance of actin cytoskeleton had no obvious difference. In contrast, in NaL-C_6_-treated cells, filamentous actin assembled around cell periphery, with only a few thin stress fibers located within cell body. The actin morphology in cells transfected with PKCζ FL was comparable to that in cells transfected with vector. However, PKCζ DN overexpression restored the morphology of actin stress fibers displayed by ghost-treated cells. The stress fibers became more robust and organized. This implied that NaL-C_6_ regulate the dynamics of actin cytoskeleton via PKCζ.

By staining paxillin, a focal adhesion marker, we showed that in ghost-treated cells, vector, PKCζ FL and PKCζ DN overexpression did not lead to the difference in focal adhesion distributions (Fig. 4a). They all displayed bright punctate focal adhesions which were colocalized with the end of thick stress fibers. When cells were treated with NaL-C_6_, focal adhesion stainings became dim. Small focal adhesions were visible at cell periphery and they almost disengaged with thin stress fibers. PKCζ FL transfection did not change the appearance of focal adhesions. On the contrary, PKCζ DN overexpression reverted the loss of focal adhesion complexes and thick stress fibers induced by NaL-C_6_ treatment. Focal adhesions moved from the cell periphery to cell body where they were associated with thick stress fibers. Quantitative analysis of the average focal adhesion size and number in a cell revealed that in NaL-C_6_-treated cells, PKCζ FL decreased but PKCζ DN increased the size and number of focal adhesions (Fig. 4b–c).

**Figure 5:**
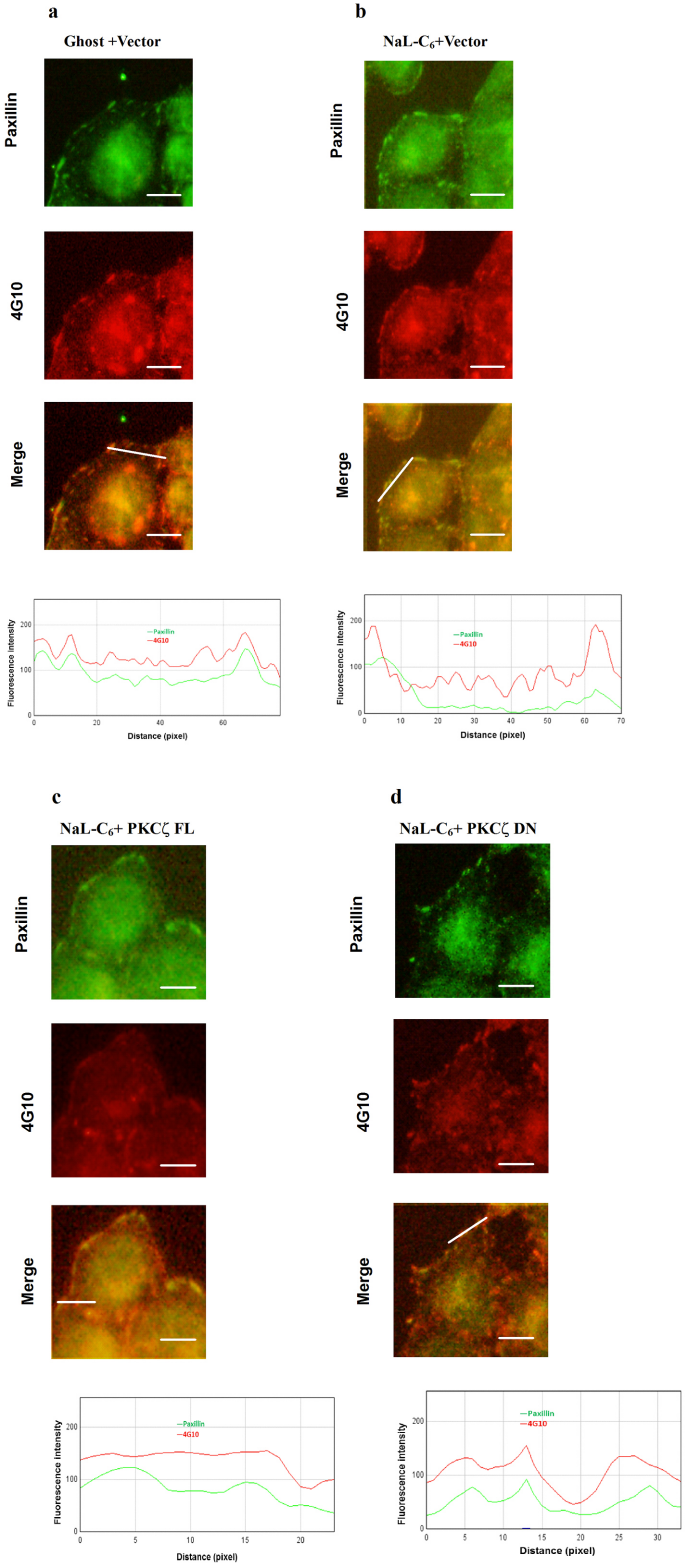
C_6_-ceramide nanoliposome-induced focal adhesion disassembly was regulated by dephosphorylation of paxillin through PKCζ activation. (a–d) MDA-MB-231 cells were transfected with vector(a–b), PKCζ FL(c), or PKCζ DN(d). Then, they were treated with ghost (a) or 20 μM ceramide nanoliposomes (b–d) before being co-stained with anti-paxillin antibody (green) and 4G10 (phospho-tyrosine specific antibody) (red).The profiles in the panel below show the fluorescence intensity patterns of focal adhesions from line scans in the merged images, which were analyzed by Image J. Bar = 5 μm.12 cells were analyzed per condition in each experiment.

Disassembly of focal adhesion complexes is usually accompanied by the dephosphorylation of paxillin molecules. To investigate whether NaL-C_6_ treatment and PKCζ disturbance may regulate the phosphorylation states of paxillin, MDA-MB-231 cells were stained with anti-paxillin antibody and 4G10, an antibody against phosphotyrosine on proteins. In ghost-treated cells, 4G10 staining appeared as large dots and colocalized with paxillin in cell body, implying that paxillins were tyrosine phosphorylated (Fig. 5a). Upon NaL-C_6_ treatment, paxillin and 4G10 dots became smaller and localized at cell periphery (Fig. 5b). The line-scanned fluorescence intensity profiles of paxillin were not correlated with those of 4G10, suggesting that paxillin was dephosphorylated. While PKCζ FL transfection did not alter the phosphorylation states of paxillin, PKCζ DN overexpression resulted in the presence of larger 4G10-stained dots in cell body where they were colocalized with focal adhesions. The line-scanned fluorescence intensity profiles of paxillin were in phase with those of 4G10 (Fig. 5c–d). This data implied that NaL-C_6_ regulated focal adhesion disassembly and paxillin dephosphorylation in a PKCζ-dependent manner.

**Figure 6:**
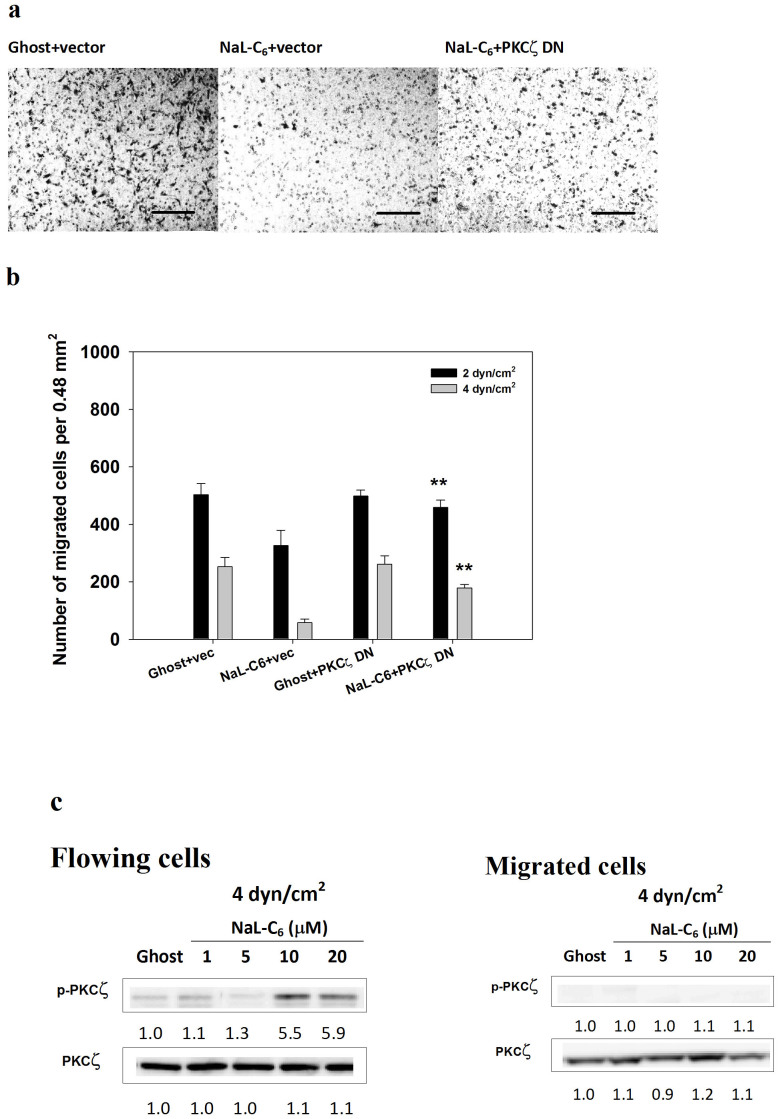
NaL-C_6_ suppressed tumor extravasation under flow conditions in a way that required PKCζ activation. (a) PKCζ DN partially restored the migratory potency of MDA-MB-231 cells which was suppressed by NaL-C_6_ as shown by images of transmigrated cells at the bottom of the filter. After 4-hr flow migration experiments at a shear stress of 4 dyn/cm^2^, chambers were dissembled and filters were stained. Images were taken from 10× objective. Ghost+vector: cells were transfected with empty vector and treated with ghost nanoliposomes; NaL-C_6_+vector: cells were transfected with empty vector and treated with C_6_-ceramide nanoliposomes; NaL-C_6_+ PKCζ DN: cells were transfected with PKCζ DN constructs and treated with C_6_-ceramide nanoliposomes. (b) PKCζ DN rescued NaL-C_6_-suppressed MDA-MB-231 migration at a shear stress of 2 or 4 dyn/cm^2^. Vector or PKCζ DN-transfected MDA-MB-231 cells were treated with ghost or 20 μM C_6_-ceramide nanoliposomes for 30 min. Then, cells were introduced into flow migration chamber together with 1.5 mg/ml fibrinogen. The number of cells migrated after 4 hr assay was measured. Results were expressed as mean ± SEM. n = three replicates.*p < 0.05 compared with NaL-C_6_+vector.(c) The lack of migratory potency of MDA-MB-231 cells was correlated with phosphorylation of PKCζ. MDA-MB-231 cells were treated with various doses of C_6_-ceramide nanoliposomes or ghost nanoliposomes for 30 min prior to flow migration experiments. After flow migration at 4 dyn/cm^2^ for 4 hr, flowing cells were collected from the circulation loop and migrated cells were trypsinzed. Flowing and migrated cells were subjected to western blotting analysis of phospho-PKCζ and total PKCζ. Data represent three replicates.

At a shear stress of 4 dyn/cm^2^, NaL-C_6_ administration abrogated cell migration ability with only a small number of cells migrating through the human umbilical vascular endothelial cell (HUVEC) monolayer (Fig. 6a *middle* vs *left*). PKCζ DN eliminated the inhibitory effect of NaL-C_6_ on cell migration (Fig. 6a *right* vs *middle*). At shear stresses of 2 and 4 dyn/cm^2^, PKCζ DN overexpression rescued NaL-C_6_-suppressed cell extravasation (*p* < 0.01). To further determine the role of PKCζ in regulating cell migration, we analyzed PKCζ phosphorylation levels in cells remaining in circulation and undergoing transmigration after 4-hr flow migration assays. As shown in Fig. 6c, nanoliposomal C_6_-ceramide upregulated PKCζ phosphorylation levels in flowing cells in a dose-dependent manner. In contrast, PKCζ phosphorylation in migrated cells was unresponsive to up to 20 μM NaL-C_6_ treatment. These results implied that PKCζ participated in NaL-C_6_-regulated cell migration in flow.

### PI3K inhibition rescued tumor migration suppressed by acute C_6_ nanoliposome treatment

**Figure 7:**
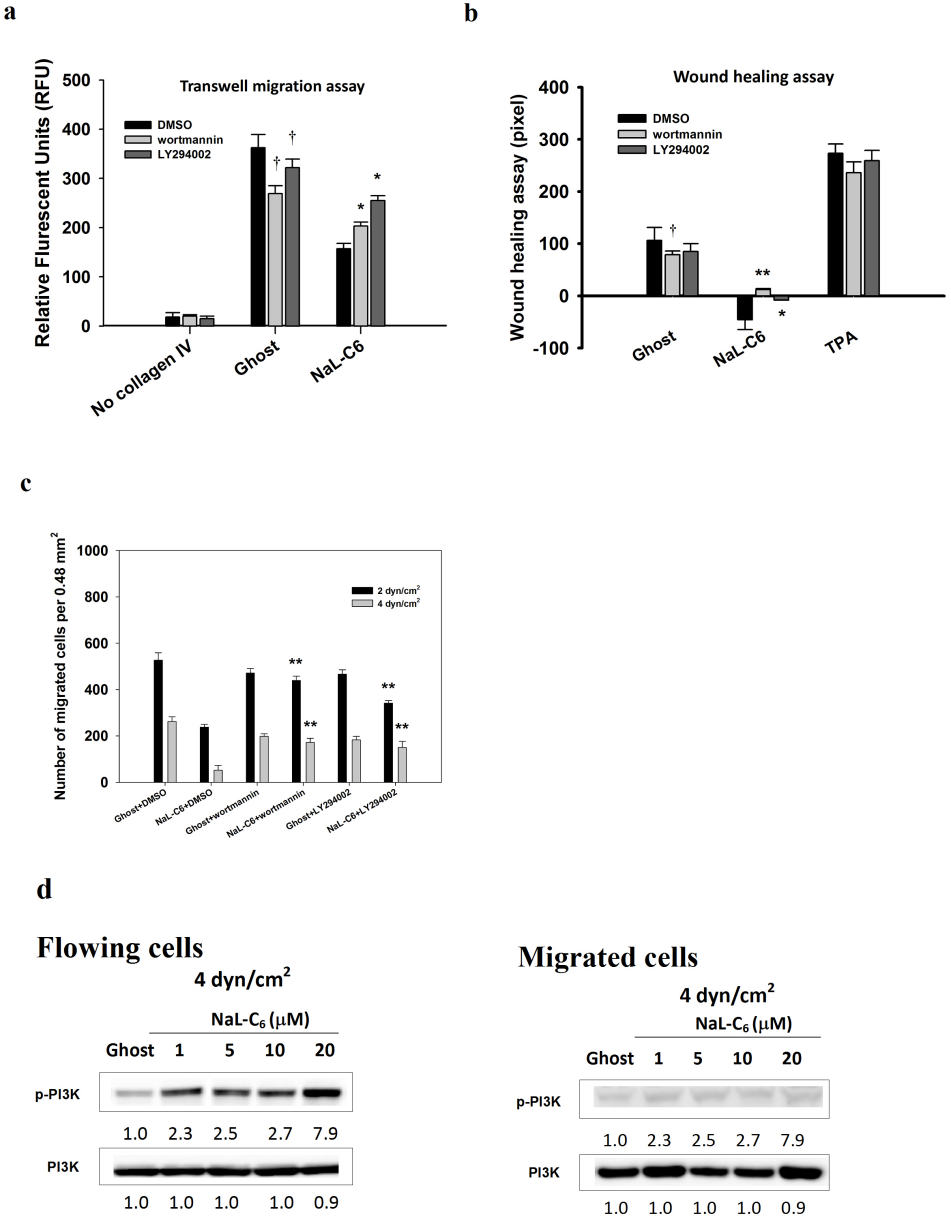
PI3K activation was required for NaL-C_6_-suppressed cell static migration and transmigration in flow. (a–c) MDA-MB-231cells were incubated with DMSO, 500 nM wortmannin or 10 μM LY294002 for 30 min before being treated with ghost or 20 μM C_6_-ceramide nanoliposomes or 200 nM TPA for 30 min. The migration of treated MDA-MB-231cells was assessed by transwell migration assay (a), wound healing assay (b) and flow migration assay (c). Results were expressed as mean ± SEM. n = three replicates. (a) *p < 0.05 compared with NaL-C_6_+DMSO; †*p* < 0.05 compared with NaL-C_6_+DMSO. (b) *p < 0.05, **p < 0.01 compared with NaL-C_6_+DMSO; †*p* < 0.05 compared with ghost+DMSO. (c) **p < 0.01 compared with NaL-C_6_+DMSO.(d) The lack of migratory potency of MDA-MB-231 cells was correlated with phosphorylation of PI3K. MDA-MB-231 cells were treated with various doses of C_6_-ceramide nanoliposomes or ghost nanoliposomes for 30 min prior to flow migration experiments. After flow migration at 4 dyn/cm^2^ for 4 hr, flowing cells were collected from the circulation loop and migrated cells were trypsinzed. Flowing and migrated cells were subjected to western blotting analysis of phospho-PI3K and total PI3K. Data represent three replicates.

To determine the role of PI3K in NaL-C_6_-induced suppression of cancer migration, we assessed transwell migration of MDA-MB-231 cells which received PI3K inhibitors, wortmannin and LY294002. Wortmannin and LY294002 significantly attenuated the transmigration of ghost-treated cells (Fig. 7a). Nevertheless, NaL-C_6_-treated cells receiving wortmannin or LY294002 migrated more vigorously than those receiving DMSO. Wortmannin and LY294002 increased the amounts of migrated cells by 29% and 64%, respectively. In addition, wortmannin and LY294002 promoted the sealing of the wound for NaL-C_6_-treated cells (Fig. 7b). Of note, wortmannin which is a more potent inhibitor for PI3K had a larger impact on cell motility than LY294002. Wortmannin exposure completely reverted the cell contraction phenotype induced by NaL-C_6_ treatment and promoted cell longitudinal migration. Wortmannin or LY294002 treatment significantly increased NaL-C_6_-suppressed MDA-MB-231 migration at shear stresses of 2 and 4 dyn/cm^2^ (Fig. 7c). At a shear stress of 4 dyn/cm^2^, PI3K phosphorylation levels were boosted by NaL-C_6_ treatment in flowing cells but not migrated cells (Fig. 7d).This suggested that PI3K was critical for ceramide nanoliposome-regulated cell migration.

### PI3K-regulated PKCζ phosphorylation and PKCα dephosphorylation were crucial for NaL-C_6_-suppressed cancer migration

**Figure 8:**
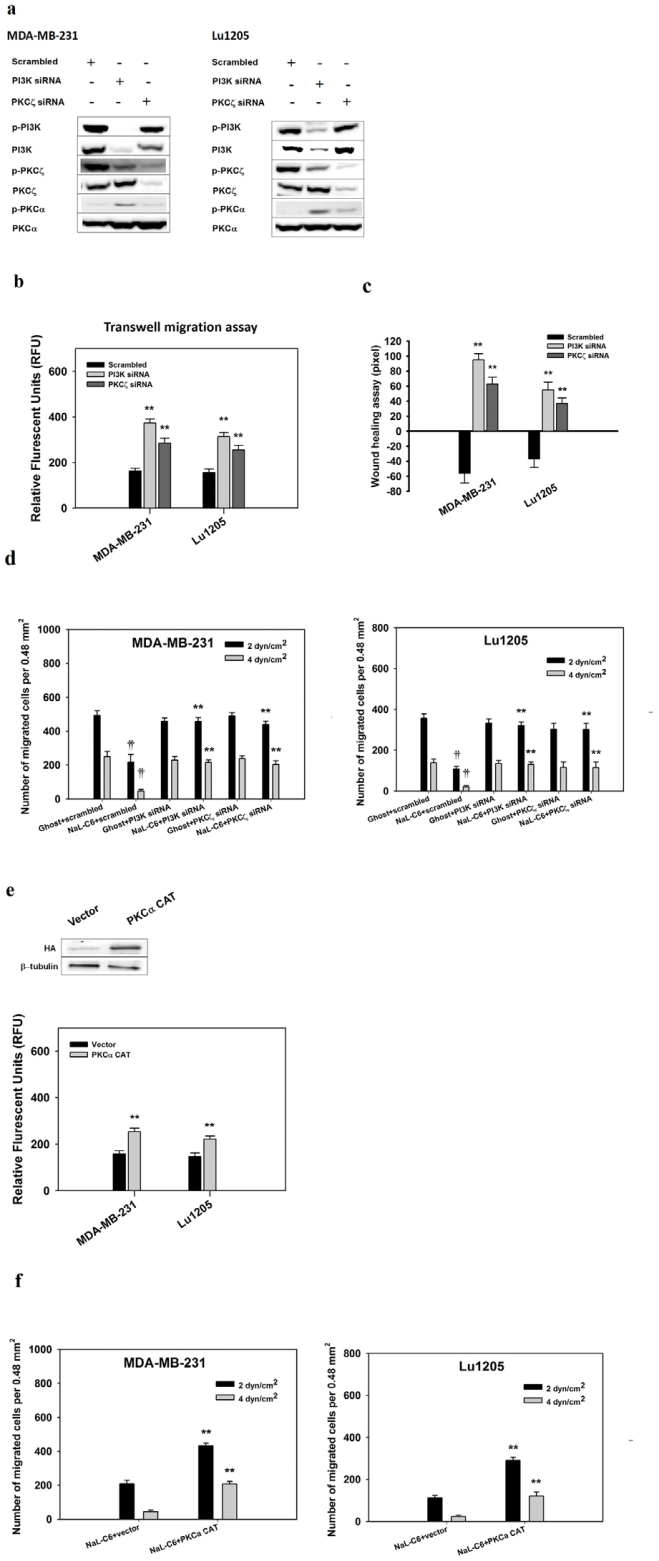
PI3K was activated upstream of PKCζ and PKCα to initiate a signaling cascade to suppress MDA-MB-231 or Lu1205 migration. (a) siRNA targeting PI3K reduced PKCζ phosphorylation and increased PKCα phosphorylation. MDA-MB-231 or Lu1205 cells were transfected with scrambled siRNA, PI3K siRNA or PKCζ siRNA before being treated with 20 μM C_6_ nanoliposome for 30 min. Subsequently, the cells were subject to Western blotting analysis of phosphorylated and total PI3K, PKCζ, and PKCα levels. PI3K, PKCζ and PKCα siRNAs exhibited >90% knockdown efficiencies. Data represent three replicates. (b–c) PI3K or PKCζ knockdown rescued NaL-C_6_-suppressed MDA-MB-231 and Lu1205 static transwell migration (b) and wounding healing potentials (c). MDA-MB-231 or Lu1205 cells were transfected with scrambled siRNA, PI3K siRNA or PKCζ siRNA before being treated with 20 μM C_6_ nanoliposome for 30 min. Results were expressed as mean ± SEM. n = three replicates. **p < 0.01 compared with scrambled. (d) PI3K or PKCζ knockdown rescued NaL-C_6_-suppressed MDA-MB-231 and Lu1205 migration at a shear stress of 2 or 4 dyn/cm^2^. Scrambled, PI3K or PKCζ siRNA-transfected MDA-MB-231or Lu2105 cells were treated with ghost or 20 μM C_6_-ceramide nanoliposomes for 30 min. Then, cells were introduced into flow migration chamber together with 1.5 mg/ml fibrinogen. The number of cells migrated after 4-hr assay was measured. Results were expressed as mean ± SEM. n = three replicates. **p < 0.01 compared with NaL-C_6_+scrambled; ††*p* < 0.01 compared with ghost+scrambled. (e–f) PKCα CAT restored NaL-C_6_-suppressed MDA-MB-231 and Lu1205 static transwell migration (e) and flow migration (f). MDA-MB-231 or Lu1205 cells were transfected with vector or PKCα CAT. The transfection efficiency was probed by western blotting with anti-HA antibody. Then, cells were treated with 20 μM C_6_-ceramide nanoliposomes for 30 min before being assessed for transwell (e) and flow migration abilities (f). **p < 0.01 compared with NaL-C_6_+vector.

To further verify the role of PI3K and PKCζ in regulating cell migration, MDA-MB-231 and Lu1205 cells were transfected with PI3K or PKCζ siRNA. PI3K and PKCζ siRNA effectively knocked down target gene expressions (Fig. 8a). PI3K knockdown reduced the phosphorylation level of PKCζ and elevated phosphorylation level of PKCα in both NaL-C_6_-treated MDA-MB-231 and Lu1205 cells. This may suggest that PI3K was activated upstream of PKCζ and PKCα in response to acute NaL-C_6_ treatment.

PI3K or PKCζ knockdown rescued MDA-MB-231 and Lu1205 transwell migration, wound healing and flow migration potencies (Fig. 8b–d). At 4 dyn/cm^2^, PI3K and PKCζ knockdown increased MDA-MB-231 cell migration by 4.7- and 4.3-fold, respectively. PKCα is known to regulate myosine light phosphorylation and actin contraction, thereby promoting cell migration. Since PKCα phosphorylation was downregulated in response to NaL-C_6_ treatment, we hypothesized that PKCα deactivation was also required for suppression of cell migration. To test the hypothesis, we transfected cells with constitutively active PKCα (PKCα CAT). PKCα CAT overexpression enhanced MDA-MB-231 and Lu1205 transwell migration and flow migration capacities (Fig. 8e–f).

### Integrin α_v_β_3_ affinity modulation was involved in PKCζ and PI3K-dependent suppression of migration by NaL-C_6_

**Figure 9:**
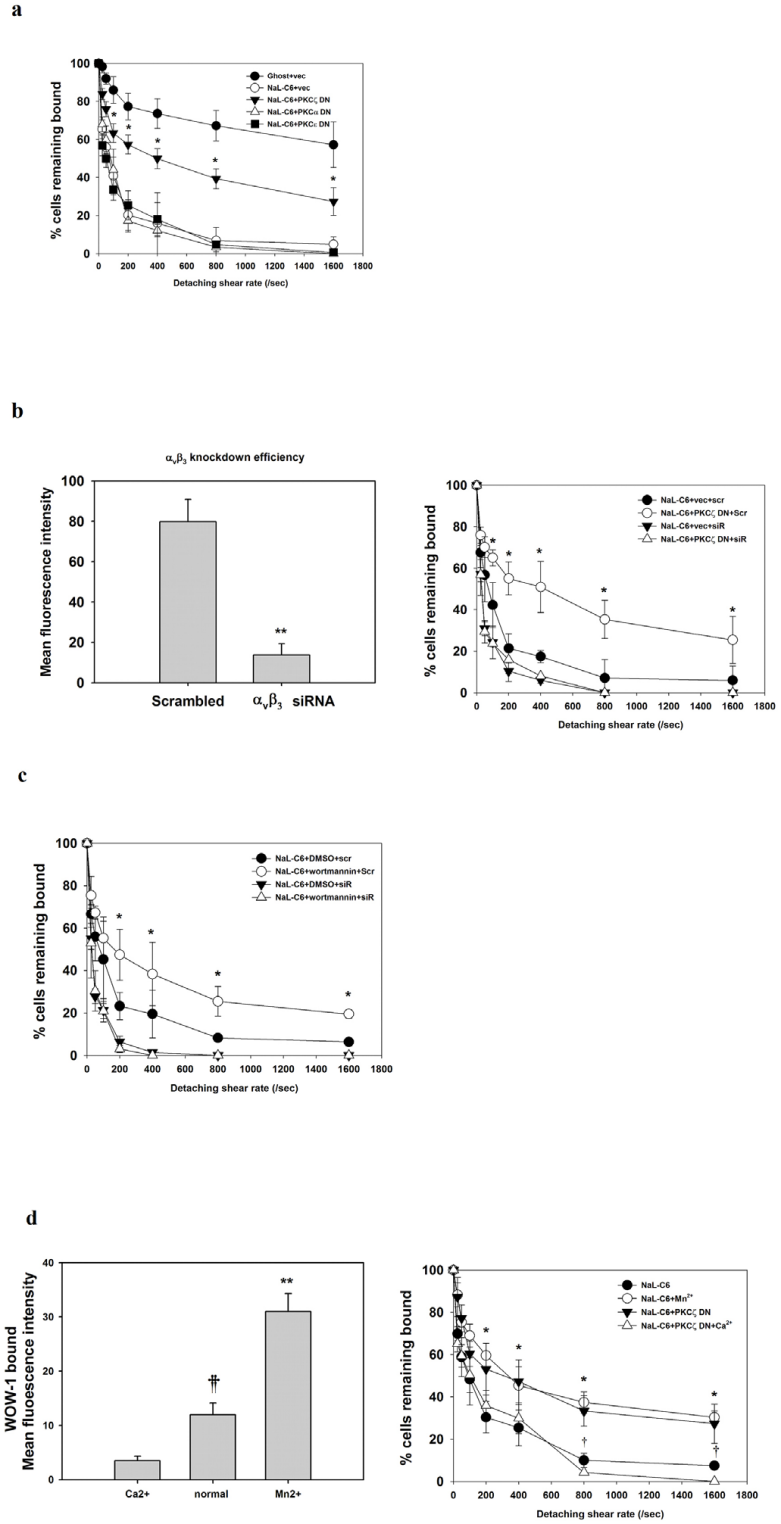
Loss of integrin α_v_β_3_-mediated shear-resistant adhesion was involved in suppressive effect of NaL-C_6_ on tumor migration via PKCζ and PI3K. (a) PKCζ DNbut not PKCα DN and PKCε DN conferred NaL-C_6_-treated cells shear-resistant adhesive capacity. Fibrinogen was coated as substrate, before the parallel plate flow chamber was assembled. Transfected MDA-MB-231 cells were settled onto fibrinogen for 7 min prior to initiation of the assays. Step-load shears (0, 50, 100, 200, 400, 800, and 1600 sec^-1^) were applied to attached cells. The percentage of cells remaining bound to fibrinogen after each shear step was determined (expressed as % cells remaining bound). Results were expressed as mean ± SEM from three independent experiments. *p < 0.05 compared with NaL-C_6_+vector at each shear rate. (b–c)Silencing integrin α_v_β_3_ by siRNA compromised the ability of PKCζ DN (b) or PI3K inhibitors (c) to rescue cell adhesion. Scrambled or integrin α_v_β_3_-targeting siRNA was introduced into MDA-MB-231 cells which were transfected with empty vector or PKCζ DN constructs(b) or incubated with DMSO, 500 nM wortmannin or 10 μM LY294002 (c). These cells were treated with 20 μM C_6_ nanoliposome for 30 min before being injected into parallel plate chamber for cell detachment assays. The percentage of cells remaining attached to fibrinogen after each shear step was determined (expressed as % cells remaining bound). Results were expressed as mean ± SEM from three independent experiments.*p < 0.05 compared with NaL-C_6_+vector+scramble at each shear rate. vec, vector; scr, scrambled siRNA; siR, integrin α_v_β_3_ siRNA. (b) Modulation of integrin α_v_β_3_ affinity was required for NaL-C_6_-mediated and PKCζ-dependent cell adhesion weakening. The ability of MDA-MB-231 cells to bind to ligand-mimetic antibody Fab WOW-1 (10 ug/ml)in the presence or absence of 1 mM CaCl_2_ or 250 μM MnCl_2_ were analyzed by flow cytometry. The mean fluorescence intensity of staining was measured from three experiments. Results were expressed as mean ± SEM. **p < 0.01 compared with normal; ††*p* < 0.01 compared with calcium. Untransfected or PKCζ DN-transfected MDA-MB-231 cells were incubated with 20 μM C_6_ nanoliposome for 30 min before being subject to cell detachment assay in the presence or absence of 1 mM CaCl_2_ or 250 μM MnCl_2_. *p < 0.05 compared with C_6_; †*p* < 0.05 compared with NaL-C_6_+PKCζ DN.

Cell attachment to endothelium and development of shear-resistant bonds were critical for tumor extravasation in flow. To assess whether the suppression of the transmigration in flow by NaL-C_6_ was caused by disruption of cell firm adhesion, we employed cell detachment assay. To conduct this assay, fibrinogen was coated as substrate in petri dish prior to parallel plate chamber assembly. MDA-MB-231 cells treated with NaL-C_6_ for 30 min were allowed to settle onto the coated fibrinogen before step-load shears were exerted. 20 μM NaL-C_6_ exposure reduced the number of bound cells with increasing shear rate (0 ~ 1600 sec^−1^) (Fig. 9a). In contrast to PKCαDN and PKCε DN, PKCζ DN transfection rescued the suppression of cell adhesion by NaL-C_6_ at each shear rate (Fig. 9a).

Since MDA-MB-231 and Lu1205 cells express integrin α_v_β_3_ which can form bonds with fibrinogen and promote tumor firm adhesion in flow, we next investigated the effect of integrin α_v_β_3_ siRNA knockdown on cell shear-resistant adhesion. Compared with scrambled siRNA control, integrin α_v_β_3_ siRNA knockdown significantly reduced cell adhesion (NaL-C_6_+vec+scr vs NaL-C_6_+vec+siR) (Fig. 9b). In the cells which were depleted of integrin α_v_β_3_ with siRNA, PKCζ DN transfection failed to restore cell adhesion ability suppressed by NaL-C_6_ (NaL-C_6_+vec+siR vs NaL-C_6_+PKCζ DN+siR). PI3K inhibition by wortmannin considerably increased the amounts of bound cells compared with DMSO (Fig. 9c). Integrin α_v_β_3_ knockdown abrogated the effect of wortmannin on cell adhesion.

Integrin affinity is regulated by divalent cations. Addition of Mn^2+^ or removal of Ca^2+^ results in increased ligand-binding affinity and adhesiveness of α_v_β_3_ integrin^37^,^38^. WOW-1 antibody which specifically recognizes α_v_β_3_ activation-epitope was used to probe integrin binding affinity modulated by ions^25^. In the presence of Mn^2+^, WOW-1 binding increased by 2.5-fold, while in the presence of Ca^2+^, WOW-1 binding decreased by 3-fold (Fig. 9d). Mn^2+^ restored MDA-MB-231 shear-resistant adhesion suppressed by NaL-C_6_. On the other hand, Ca^2+^ addition significantly reduced the adhesion of PKCζ DN-transfected and NaL-C_6_-treated cells. These results implied that affinity modulation of integrin α_v_β_3_ was required for PI3K and PKCζ-dependent cell adhesion weakening mediated by acute NaL-C_6_ treatment.

## Discussion

**Figure 10:**
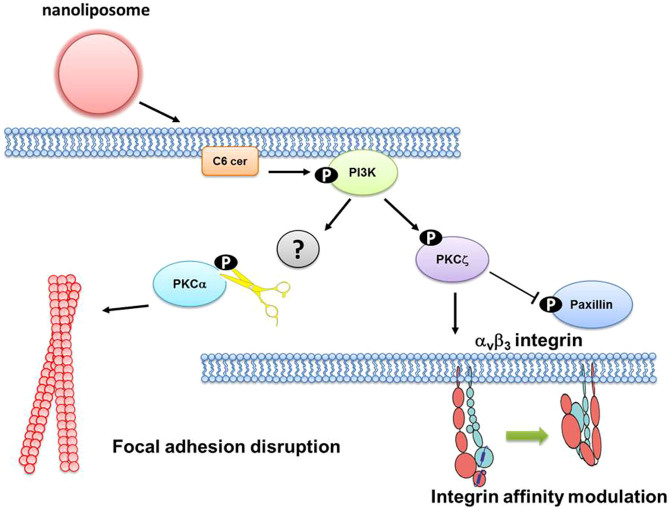
Proposed mechanism of C_6_ nanoliposome-mediated suppression of tumor migration.

Cancer metastasis is highly coordinated, multistep process, involving tumor undergoing epithelial-mesenchymal transition, traveling in blood stream, lodging onto vascular endothelium and extravasation^14^. By using wound healing, transwell migration and flow migration assays, we revealed that short-term C_6_ nanoliposome treatment suppressed melanoma and breast cancer migration without inducing cell apoptosis. In addition, we found that NaL-C_6_ initiates a very distinct signaling pathway to suppress cancer extravasation under both static and hydrodynamic conditions (Fig. 10). Pharmacological inhibition of PI3K, transient expression of dominantly negative PKCζ construct, and siRNA knockdown of PI3K or PKCζ, suppressed cell adhesion and migration. PI3K is activated by NaL-C_6_ to mediate phosphorylation of PKCζ and dephosphorylation of PKCα. PKCζ plays double roles in regulating cell migration. On one hand, it induces cytoskeletal architecture disruption, paxillin dephosphorylation and focal adhesion disassembly; on the other hand, it reduces the affinity of integrin α_v_β_3_, thereby weakening cell adhesion in flow. The inhibitory activity of PKCζ and PI3K in NaL-C_6_-treated cells distinguish them from pro-mitogenic and pro-migratory activities ascribed to conventional and novel PKC isoforms and PI3K/Akt axis.

In the current study, C_6_-ceramide was delivered in a nanoliposomal form which presents as an effective way of reducing the hydrophobicity of the ceramide and increasing its membrane transport^2^. Upon liposomal administration, ceramide is likely to be inserted into membrane lipid bilayer and localized to structured microdomain where it can be associated with signaling proteins and initiate intracellular signaling cascades^3^. In the current study, we found that in contrast to nonliposomal ceramide, liposomal ceramide more effectively suppressed tumor migration. The roles of sphingolipid metabolites in regulating cell migration remain elusive. Long-chain ceramide C_16_ was reported to enhance mouse embryonic stem cell migration in a dose- and time-dependent manner^39^. On the other hand, sphingosine-1-phosphate which can be converted to ceramide via sphingosine-1-phosphate phosphatase and ceramide synthase inhibited chemotactic motility of breast cancer cells^40^. Furthermore, C_2_ ceramide was reported to suppress cancer invasiveness through downregulating MMP-2 expression^41^. In the current study, the migration-suppressive effect of nanoliposomal C_6_ was a consequence of activating of PKCζ and PI3K. It was likely that short- and long-term ceramide treatments orchestrated different signaling pathways to mediate migratory inhibition and apoptosis. Long-term ceramide treatment initiated a pro-apoptotic pathway involving inactivation of Akt^2^, while acute ceramide treatment recruit and activate PI3K. In the current study, we focused on the 30-min acute effect of C_6_ nanoliposome. This is because upon adhesion to endothelium, tumor rapidly extravasates with a time period of less than 1 hr^8^,^9^. The time duration for circulating tumor to interact with nanoliposome is short, especially in face of shear force. Thus, studying tumor responses to acute, rather than prolonged, NaL-C_6_ treatment may be a more accurate reflection of physiological conditions.

In agreement with previous studies, the current study showed that NaL-C_6_ treatment decreased membrane localization of phosphorylated PKCα^42^. PKCα is known to regulate myosin light chain phosphorylation and actomyosin-mediated cell migration. PKCα activation was critical for focal adhesion formation and integrin-mediated cell migration^43^. Dephosphorylation of PKCα by nanoliposomal ceramide may decrease myosin light chain phosphorylation, preventing cell migration. PKCζ was shown to participate in cell adhesion and migration. By using inhibitors, PKCζ was suggested to promote epidermal growth factor-mediated breast cancer chemotaxis^44^. PKCζ in neutrophils regulated chemoattractant-induced actin assembly, integrin-dependent adhesion and cell migration^45^. PKCζ also participated in the migration of CD34+ progenitor cells^46^. In the current study, PKCζ suppressed tumor migration in response to NaL-C_6_ treatment, implying that different stimuli may confer PKC different functionalities.

PI3K is a family of lipid kinases which transduce signals from various growth factors and cytokines by generating phospholipids^47^. In response to stimulation, PI3K is recruited to the membrane by direct interaction of its p85 subunit with tyrosine phosphate motifs on receptors. The activated p110 catalytic subunit generates phosphatidylinositol-3,4,5-triphosphate, which serves as docking sites for several signaling proteins. PI3K was suggested to participate in integrin α_v_β_3_-mediated melanoma migration, by inducing actin stress fiber formation and enhancing integrin α_v_β_3_ avidity^48^. Activation of PI3K by Cdc42 and Rac1 alters actin organization, leading to increased motility and invasiveness. In the current study, activation of PI3K was negatively correlated with cell migration, suggesting an inhibitory role of PI3K on the migration of nanoliposomal ceramide-treated cells.

Temporal and spatial regulation of cytoskeletal organization and focal adhesion formation plays an essential role in cell migration. It was evident that nanoliposomal ceramide administration had an impact on both cell motility and receptor-mediated adhesion. Cell motility is driven by actin-based protrusion at cell's leading edge^49^. Previous studies showed that mutations in paxillin phosphorylation sites reduced focal adhesion formation^50^. It was also suggested that phosphorylation of paxillin promotes the association of unphosphorylated focal adhesion kinase with paxillin at newly growing focal contact sites, thereby promoting cell motility and migration^51^,^52^. Paxillin association with stress fiber at adhesion sites may be critical for transmitting propulsive forces and serve as traction points over which cell moves^49^. Therefore, cancer cell migration speed is a function of cell adhesion strength, as regulated by focal adhesion size and number. Highly polymerized cytoskeleton exerts tension on adhesion sites and allows retraction of cell, increasing the migration speed of cells. It is likely that ceramide suppresses cell motility by mediating PKCζ-dependent stress fiber disassembly and dephosphorylation of paxillin.

To undergo extravasation in blood vessel, cancer cells must tether and firmly adhere to endothelium. Different molecular constituents are required for multistep cell adhesion^53^. By generating shear-resistant bonds whose dissociation constants are regulated by flow force, integrin mediates firm adhesion of cells^54^,^55^. Cell detachment assay revealed that silencing integrin α_v_β_3_ compromised the abilities of PI3K inhibitors and PKCζ DN to rescue cell adhesion. Therefore, it is likely that the downstream target of PI3K and PKCζ is integrin-mediated sustained cell adhesion. Integrins are known to exist in distinct activation states, which exhibit different affinities for ligands^37^,^56^. In general, integrin activation controls cell adhesion. Such control is particularly important in the vasculature, where dynamic flow physically opposes cell attachment. Cell adhesion strengthening through α_v_β_3_ integrin activation enhances cell transmigration in flow. The affinity status of integrin α_v_β_3_ was regulated by “inside-out” signaling^31^. Studies indicated that integrin α_v_β_3_ activation status regulated breast cancer migration and metastasis^25^,^57^. It was suggested that PKCζ played a role in integrin activation in response to chemoattractant stimulation^45^. By using integrin affinity regulating ions, we established a causal relationship between NaL-C_6_-mediated suppression of cancer metastasis and the deactivation of integrin α_v_β_3_. PI3K and PKCζ might directly or indirectly decrease integrin α_v_β_3_ affinity to compromise cell shear-resistant adhesion^58^.

In conclusion, acute C_6_-ceramide nanoliposome treatment resulted in a significant reduction in melanoma and breast cancer migration and metastasis. Nanoliposomal ceramide-mediated suppression of cell migration under static and flow conditions was dependent on PI3K and PKCζ activation. Cytoskeletal remodeling, focal adhesion disassembly and integrin α_v_β_3_ affinity modulation were involved in this process. Understanding the molecular mechanisms and intracellular pathways downstream of nanoliposomal ceramide may facilitate the development of therapeutic strategies to prevent tumor metastasis.

## Methods

### Cell culture and reagent

DOPE, 1,2-distearoyl-sn-glycero-3-phosphocholine (DSPC), dioleoylphosphatidylcholine (DOPC), D-*erythro*-hexanoyl-sphingosine (C_6_-ceramide), 1,2-distearoyl-sn-glycero-3-phosphoethanolamine-*N*-[methoxy polyethylene glycol-2000], and *N*-octanoyl-sphingosine-1-[succinyl(methoxypolyethylene glycol-750)] (PEG(750)-C_8_) were purchased from Avanti Polar Lipids (Alabaster, AL). 12-O-tetradecanoylphorbol-13-acetate (TPA), anti-PKCα, anti-PKCε, anti-PKCζ, anti-phospho-PKCα/βII (Thr638/641), anti-phospho-PI3 Kinase p85 (Tyr458)/p55 (Tyr199) and anti-PI3K antibodies were purchased from Cell Signaling Technology (Massachusetts, MA). Anti-HA, anti-paxillin, anti-phospho-PKCζ (Thr 410) and anti-phospho-PKCε (Ser729) were purchased from Santa Cruz (Dallas, TX). Mouse IgG anti-human α_v_β_3_ (anti-CD51/61, clone 23C6), CD44H, VLA-4 and ICAM-1 (clone BBIG-I1) were purchased from R&D Systems (Minneapolis, MN). 4G10 (anti-phosphotyrosine antibody) was obtained from Millipore (Billerica, MA). Rhodamine-phalloidin, 4',6-diamidino-2-phenylindole, dihydrochloride (DAPI), calceim-AM and mouse anti-human mAbs against Mac-1 (anti-CD11b) and LFA-1 (anti-CD11a) were purchased from Invitrogen (Carlsbad, CA). Mouse anti-human mAbs directed to sialyl-Le^x^ and sialyl-Le^a^ were purchased from Calbiochem (San Diego, CA). 24-well transwell devices with polycarbonate membrane were purchased from Corning (Corning, NY). TransIT 2020 was purchased from Mirus (Mirus Bio LLC, Madison, WI). Super Signal West pico chemiluminescence reagent and ImmunoPure antibody goat anti-mouse IgG horseradish peroxidase were from Thermo Scientific (Rockford, IL). Wortmannin, LY294002, recombinant fibrinogen, TNF-α bovine serum albumin (BSA) were purchased from Sigma-Aldrich (St. Louis, MO).

Lu1205 and MDA-MB-231 cells were obtained from American Type Culture Collection (Manassas, VA). Cells were cultured in DMEM/F12, supplemented with 10% fetal bovine serum (FBS). HUVECs (American Type Culture Collection) were maintained in F12K media supplemented with 50 μg/ml heparin(Mallinckrodt Baker, Inc), 30 μg/ml endothelial growth factor (Sigma Aldrich, Shanghai, China) and 10% FBS. Cells in passage number of 5–10 were used for experiments. FBS, DMEM/F12, F-12K, non-essential amino acids, sodium pyruvate, penicillin/streptomycin and l-glutamine were all purchased from GIBCO (Gaithersburg, MD).

#### Flow cytometry

To examine adhesion molecule expression, cells were incubated with saturating concentrations of primary mAbs directed against specific adhesion molecules (ICAM-1, VLA-4, sialyl-Le^a/x^, α_v_β_3_, LFA-1, Mac-1 and CD44) in DPBS containing 1% BSA for 20 min at 4°C and then washed twice. After an additional 30 min incubation with tetramethylrhodamine isothiocyanate (TRITC)-conjugated goat anti-mouse Fab_2_ fragment (1 μg/10^6^ cells; Jackson Immuno Research, West Grove, PA) at 4°C, the cells were washed twice and fixed with 2% formaldehyde and analyzed by a GUAVA flow cytometry (GUAVA technologies, Burlingame, CA) and FACSCalibur (BD, San Jose, CA, USA). To measure binding of the ligand mimetic antibody Fab WOW-1(generously donated by Dr. Sanford Shattil from The Scripps Research Institute), cells were incubated for 30 min with 10 μg/ml Fab in 135 mM NaCl, 2.7 mM KCl, 3.3 mM NaH_2_PO_4_, 3.8 mM HEPES, and 1 mg/ml BSA, pH 7.4, with or without 250 μM MnCl_2_ or 1 mM CaCl_2_. Cells were washed and incubated with TRITC anti-mouse IgG and analyzed on FACSCalibur flow cytometry.

#### Plasmid transfection

MDA-MB-231 or Lu1205 cells were transiently transfected with empty pCDNA3 expression vector, PKCζ FL, PKCζ DN (agenerous gift from Dr. J. Moscat), constitutively active PKCα (PKCα CAT), dominantly negative PKCαPKCαDN), or dominantly negative PKCεPKCε DN) constructs. 2.5 μg plasmids were transfected into cells with 7.5 μg Mirus TransIT 2020. The transfection efficiency was detected with western blotting or fluorescence staining. PKCζ FL is a wildtype construct in a pCDNA3 expression vector which was cloned from PKCζ genomic sequence (GenBank accession ID: NM_002744.4). The dominant-negative mutant constructs are kinase defective mutants that contain point mutations in the catalytic domain, i.e. K281R for PKCζ, K368R for PKCα(GenBank accession ID: NM_002737.2)and K436R for PKCε (GenBank accession ID: NM_005400.2). These point mutations were generated by QuickChange II site-directed mutagenesis kit (Agilent Technologies, Palo Alto, CA).

#### Pharmacological inhibition

To inhibit PI3K, MDA-MB-231 or Lu1205 cells were treated with the following signaling inhibitors: 500 nM wortmannin or 10 μM LY294002 diluted in DMSO for 30 min at 37°C, prior to running of assays. To activate PKC, cells were treated with 200 nM TPA for 30 min.

#### siRNA and transfection

Duplexed Stealth small interfering RNAs(siRNA) towards integrin α_v_ and β_3_ were purchased from Invitrogen (Carlsbad, CA).Transfection was performed with nucleofection using an Amaxa Nucleofector (Lonza) with Solution R/program K-17. A total of 100 pmol siRNA was introduced into 1.0 × 10^6^ tumor cells. The transfection efficiency to knockdown integrin α_v_β_3_ reached 80% after 48 hr as assessed by western blotting and flow cytometry. Following siRNA introduction, cells were replated in culture dishes. siRNA sequences were: scrambled: 5′-AAUUCUCCGAACGUGUCACGUGAGA-3′; integrin α_v_:

5′-UUGAUGAGCUCAUAGACAUGGUGGA-3′; and integrin β_3_: 5′-AUAAGCAUCAACAAUGASGCUGGAGG-3′.

PKCζ siRNA (5′-GACAGACGCUUGCGCCGAGAC-3′) was synthesized by Invitrogen. PI3K siRNA SMARTpool (Thermo Scientific, Rockford, IL) which contained 4 pooled siRNA duplexes with “UU” overhang was employed to silence PI3K. MDA-MBA-231 or Lu1205 were plated in a 6-well plate and were 50–70% confluent when siRNA was introduced. Transfection was performed with 50 nM siRNA complexed with 7.5 ml RNAiMax transfection reagent. Medium was changed after 6 hrs. The transfection efficiency to knockdown PKCζ or PI3K reached 80% after 48 hrs as assessed by Western blotting.

#### Preparation of nanoliposomal ceramide

Preparation of nanoliposomal C_6_-ceramide was previously described with a slight modification. Briefly, DSPC, DOPE, C_6_-ceramide, 1,2-distearoyl-sn-glycero-3-phosphoethanolamine-N-[methoxypolyethylene glycol-2000], and PEG(750)-C_8_ were mixed in chloroform at a molar ratio of 3.75:1.75:3.5:0.70:0.70 and evaporated under vacuum in a water bath at 25°C. After complete removal of the solvent,a thin film formed. Saline was added to the lipid film and hydrated for 30 min to obtain a crude dispersion of liposomes containing 30% ceramide. The resulting solution was then homogenized by sonication for 10 min followed by filtration through a 100-nm polycarbonate membrane with Avanti Mini Extruder (Avanti Polar Lipids). Ghost liposomes were prepared in a similar manner without C_6_-ceramide. The size distributions of nanoliposomes were measured by dynamic light scattering. Zeta potentials of the nanoliposomes were determined to be within the range of −3 mV to −6.5 mV, ensuring a neutral charge on the nanoliposomes.

#### Fluorescent staining

Untransfected or transfected tumor cells were grown on cover slips before experiments. Cells were incubated with ghost nanoliposome or 20 μM nanoliposomal C_6_-ceramide for 30 min. Cells were then washed twice with DPBS and fixed with 5% formaldehyde for 10 min. Cells were permeabilized with 0.3% Triton X-100 in DPBS and blocked for 30 min with 5% BSA. Subsequently, cover slips were incubated with anti-paxillin and 4G10 for 1 hr at room temperature. This was followed by staining with Alexa 488-conjugated anti-rabbit IgG and Alexa 555-conjugated anti-mouse IgG. To image actin filaments, rhodamine-phalloidin (1:40; Invitrogen) was incubated with cells for 40 min. Finally, fluorescent staining was visualized with a fluorescence microscope (Olympus IX71). Colocalization of paxillin and actin or 4G10 was processed by ImageJ. Results were representative of three independent experiments.

#### Membrane and cytosol fractionation

After being incubated with different doses of nanoliposomal ceramide, cells were harvested on ice. Cells were washed with ice-cold DPBS, resuspended in 250 μl homogenization buffer (20 mM Tris pH 7.4, 10 mM EGTA pH 7.4, 2 mM EDTA pH 7.4, 10 mM sodium orthovanadate, 5 mM sodium fluoride, 1 mM DTT, 1 mM β-glycerophosphate, 50 nM okadaic acid, 0.02% leupeptin, 0.01% aprotinin, 0.01% trypsin-chymotrypsin inhibitor, 1 mM phenylmethylsulfonyl), and lysed by sonication three times for 30 sec. Nuclei and unbroken cells were pelleted by centrifugation at 1,000 × *g* for 10 min and supernatants were centrifuged at 120,000 × *g* for 30 min at 4°C. Supernatants (cytosolic fraction) were stored at −80°C. Pellets (membrane fractions) were resuspended in 100 μl of homogenization buffer containing 0.8% Triton X-100, sonicated once for 30 sec, and stored at −80°C. Protein concentrations of the fractions were determined using Bradford assay.

#### Western blotting

After functional assays, tumor cells were collected and rinsed with DPBS, and lysed with RIPA lysis buffer (20 mM Tris, 5 mM MgCl_2_, 1 mM PMSF, 20 mg/ml aproptonin, 10 mg/ml leupeptin, 1 mM Na_3_VO_3_, and 20 mM β-glycerophosphate). The lysates were centrifuged at 14,000 rpm for 15 min. The protein concentrations across samples were checked by Bradford method. The samples were denatured by adding SDS running buffer (0.2% bromophenol blue, 4% SDS, 100 mM Tris[pH 6.8], and 20% glycerol) and β-mercaptoethanol. The samples were analyzed by SDS-PAGE on 12% gels. After the proteins were transferred to nitrocellulose membrane, phosphorylated PKCα (Thr638), phosphorylated PKCε (Ser729), phosphorylated PKCζ(Thr410), phosphorylated PI3K p85 (Tyr458) or HA were detected with corresponding primary monoclonal antibodies(1:1000 diluted in blocking buffer) followed by HRP conjugated secondary antibodies. The labeled proteins were visualized using an enhanced chemilumenscence kit (Amersham, Arlington Heights, IL). Thereafter, membranes were stripped with stripping buffer before being reprobed with anti-PKCα, PKCε, PKCζ, PI3K or β-tubulin to ensure equal loadings.

#### Transwell migration

Transmigration was assessed by the ability of cells to migrate through a porous (8 μm) polycarbonate membrane of a transwell device (Corning, NY) towards a chemotactic cue (collagen IV). HUVECs were grown on the filter membrane of transwell inserts to form monolayer. MDA-MB-231 or Lu1205 cells were incubated with NoL-C_6_, ghost nanoliposome, or NaL-C_6_. Thereafter, equal numbers (2.5 × 10^5^) of cells were plated in the inserts with HUVEC monolayer in serum-free medium containing 1% BSA. Inserts were placed in corresponding wells of a 24-well plate with 100 mg/ml collagen IV in the bottom wells. The cells were allowed to transmigrate across the porous membrane filter for 4 hr. HUVECs and unmigrated cells at the top of the membrane filter were removed by a cotton swab while the cells at the bottom of the filter were fixed in 5% formaldehyde, and stained using calcein AM (Molecular Probes, Eugene, OR). The relative fluorescence units (RFU) for each well were determined by FLx800 Fluorescence Microplate Reader (BioTek, Winooski, VT) with 485 nm excitation and 520 nm emission.

#### Wound healing assay

MDA-MB-231 or Lu1205 cells were cultured in 6-well plates at 1 × 10^6^ cells/well as confluent monolayers. The monolayers were incubated with ghost or ceramide nanoliposomes in the absence of serum for 30 min and wounded in a line across the well with a 200-μl standard pipette tip. The wounded monolayers were then washed twice with serum-free media to remove cell debris. Photographs of a selected area of each scratch were taken at indicated time points using 40X lense Olympus IX71. Wound healing effect was determined by calculating the difference in total pixels of remaining cell-free area and initial wound area.

#### Flow migration assay

Tumor cell migration was determined using a modified 48-well chemotactic chamber consisting of top and bottom plate separated by a gasket. Prior to each experiment, a monolayer of HUVECs was grown on sterile polyvinylpyrrolidone-free polycarbonate filters (8 μm pore size; NeuroProbe, Gaithersburg, MD, USA) pre-coated with fibronectin (30 μg/ml) for 2 hr (Sigma-Aldrich, St Louis, MO, USA). Prior to the experiments, HUVEC monolayers were activated with TNF-α for 6 hr to allow adhesive molecule levels to reach maximum. The center 12 wells of bottom plate were filled with soluble chemoattractant type IV collagen (100 mg/ml diluted in DMEM with 0.1% BSA; BD Biosciences, San Jose, CA, USA) and surrounding control wells were filled with DMEM containing 0.1% BSA. Previous work has shown that human cancer cells migrate toward collagen IV acting as a chemoattractant^28^. The apparatus was assembled by laying the filter on the bottom plate, followed by a gasket and top plate. The chamber was primed with DMEM and 0.1% BSA to eliminate bubbles from the system. Then, cells were placed in the chamber in the presence of fibrinogen and subjected to a shear flow (2 or 4 dyn/cm^2^) for 4 hr in an incubator. Migrated cells were stained with Protocol Brand Hema3 solution (Fisher Scientific). Cells in 12 random selected fields were counted using an inverted microscope (Diaphot 330, Nikon) with the NIH ImageJ software.

#### Cell detachment assay

Cell detachment assay reflects receptor affinity changes and the strength of adhesion. The petri dishes coated with 2.5 mg/ml fibrinogen were assemble in parallel-plate flow chamber and mounted on the stage of an inverted phase contrast microscope Olympus IX71. Tumor cells were transfected with empty vectors or PKC mutant constructs or treated with PI3K inhibitors. After being incubated with ghost or NaL-C_6_ for 30 min, cells were perfused into flow chamber. The cell concentrations from the inlet were controlled at similar levels and the fields of view were focused on the same distance from the inlet to avoid variation of initial cell concentrations. Cells were allowed to settle on fibrinogen for 7 min, before being subject to step-load shears (0 ~ 1600 sec^−1^). Wall shear stress was increased every 10 sec. The percentage of cells remaining bound was determined. In selected experiments, cell detachment assays were conducted in presence or absence of 250 μM Mn^2+^ or 1 mM Ca^2+^.

#### AnnexinV/7-AADassay

Two-color flow cytometry with annexin-V (5 ml/sample; BD Pharmingen), and 7-amino-actinomycin D (7-AAD; 10 ml/sample; BD Pharmingen) were used to assess the degree of cellular apoptosis in cells treated with C_6_-ceramide. For each sample, 5 × 10^5^ cells were plated in triplicate in 24-well plates in 0.5 ml volume and the percentage of specific apoptosis is calculated using the following formula: Apoptosis (%) = (%Annexin-V-allophycocyanin conjugate (APC) positive in assay well – % Annexin-V-APC positive in the control well)X100/(100 – % Annexin-V-APC positive in the control well).

#### Statistical analysis

All data were obtained from at least three independent experiments and expressed by means ± SEM. Statistical significance was determined using Student's *t*-test or ANOVA. Tukey's test was used in *post hoc* analysis for ANOVA. A probability value of *p <* 0.05 or *p <* 0.01 was considered to be statistically significant.

## Author Contributions

P.Z., C.F., C.D. and Y.S. designed experiments. P.Z. and C.F. performed experiments. P.Z., C.F., C.D., Y.H., E.S. and Y.S. discussed the results and interpreted the data. P.Z., C.F. and Y.H. wrote and revised the paper.

## Supplementary Material

Supplementary Information Dataset 1

## Figures and Tables

**Table 1:** Flow cytometry analysis of adhesion molecule expression on MDA-MB-231 (1^st^ row) and Lu1205 (2^nd^ row)

Control IgG (Control IgM)	ICAM-1	VLA-4	sialyl-Le^x^	sialyl-Le^a^	α_v_β_3_	LFA-1	Mac-1	CD44H
7.3 ± 0.5 (11.3 ± 1.5)	105.1 ± 4.7*	7.8 ± 2.3	(157.5 ± 3.6)*	(131.0 ± 2.8)*	77.5 ± 1.5*	9.2 ± 2.3	8.5 ± 3.6	129 ± 5.1*
3.9 ± 0.3 (9.5 ± 2.7)	167 ± 12.8*	3.7 ± 1.0	(6.5 ± 2.5)	(5.3 ± 1.6)	55 ± 1.2*	3.2 ± 1.1	3.8 ± 1.5	178 ± 17.2*

Values are geometric mean fluorescence intensities ± SEM of three experiments using different batches of cells each time. The parentheses indicate that the antibody used to test the expressions of adhesive molecules is IgM antibody.

*p < 0.05 compared with respective control cases.
