# Supplementary material for: C6-ceramide nanoliposome suppresses tumor metastasis by eliciting PI3K and PKCζ tumor-suppressive activities and regulating integrin affinity modulation
Source: Sci Rep. 2015 Mar 20;5:9275. doi: 10.1038/srep09275 (PMC4366857; doi:10.1038/srep09275)

Supplementary Data

C_6-ceramide nanoliposome suppresses tumor metastasis by eliciting PI3K and PKCζ tumor-suppressive activities and regulating integrin affinity modulation

Pu Zhang, Changliang Fu, Yijuan Hu, Yang Song, Erqun Song

Figure 2 a

MDA-MB-231

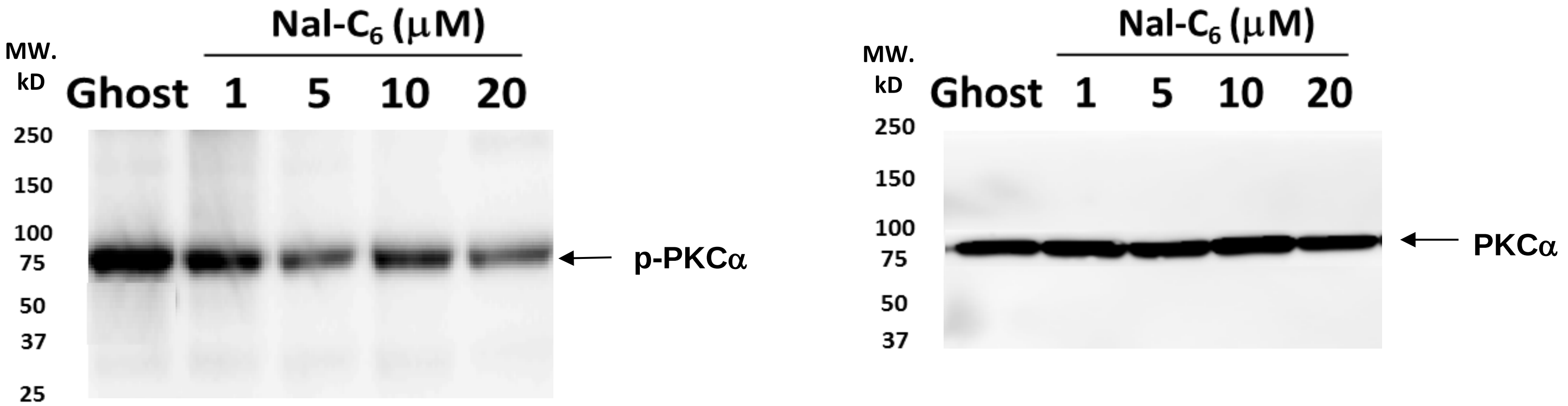

Lu1205

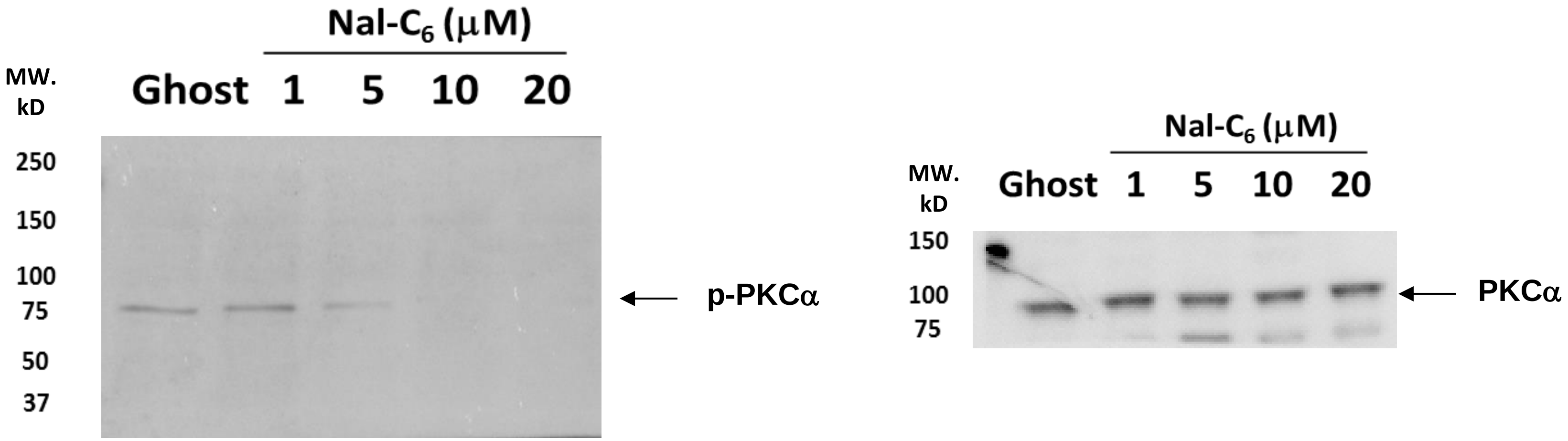

Figure 2 b

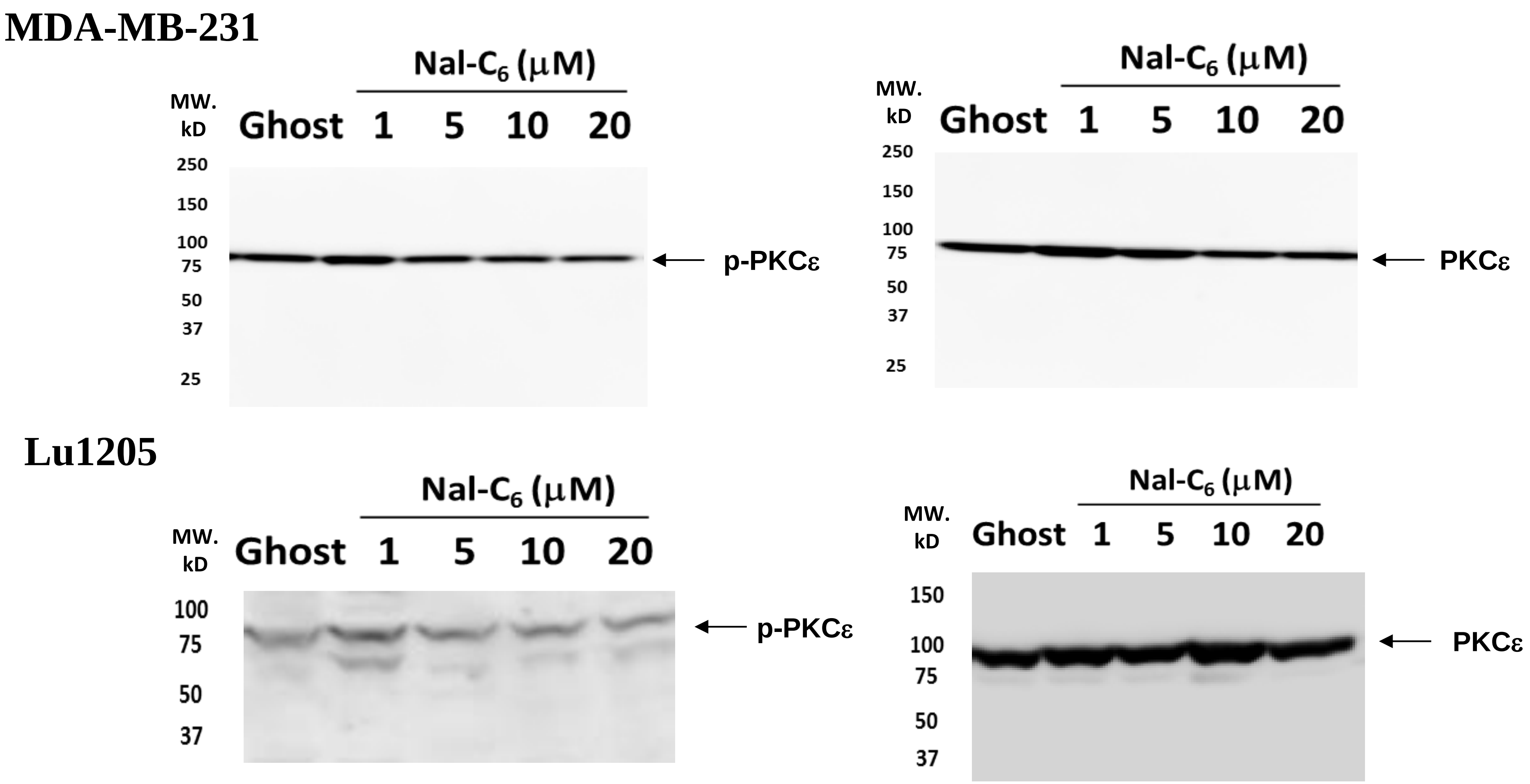
MDA-MB-231
Nal-C6 (μM)
MW. kD
Ghost 1 5 10 20
250
150
100
75
50
37
25
p-PKCε
Nal-C6 (μM)
MW. kD
Ghost 1 5 10 20
250
150
100
75
50
37
25
PKCε
Lu1205
Nal-C6 (μM)
MW. kD
Ghost 1 5 10 20
100
75
50
37
p-PKCε
Nal-C6 (μM)
MW. kD
Ghost 1 5 10 20
150
100
75
50
37
PKCε

Figure 2 c

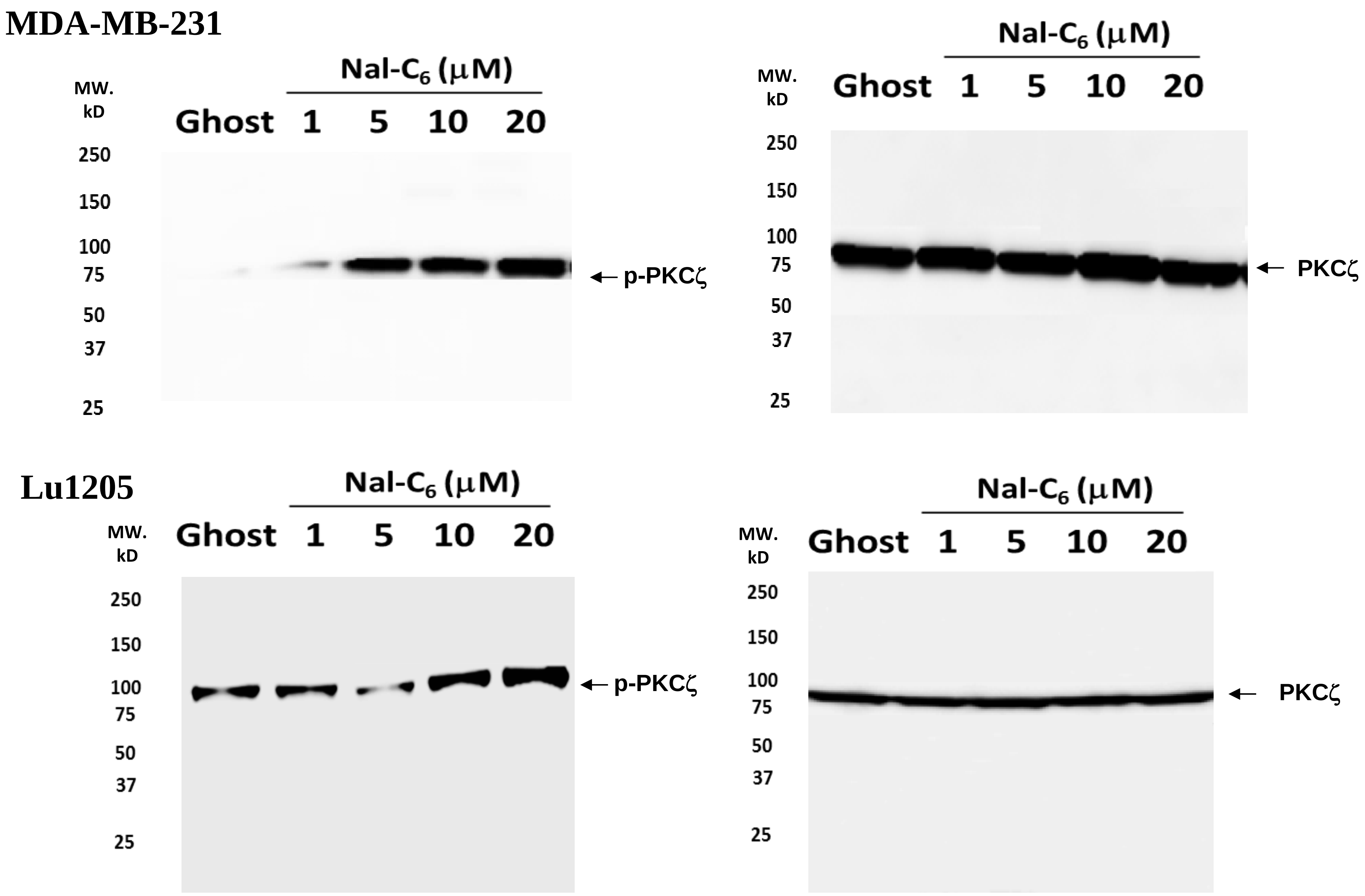
MDA-MB-231
Nal-C6 (μM)
MW. kD
Ghost 1 5 10 20
250
150
100
75
50
37
25
← p-PKCζ
← PKCζ
Lu1205
Nal-C6 (μM)
MW. kD
Ghost 1 5 10 20
250
150
100
75
50
37
25
← p-PKCζ
← PKCζ

Figure 2 d

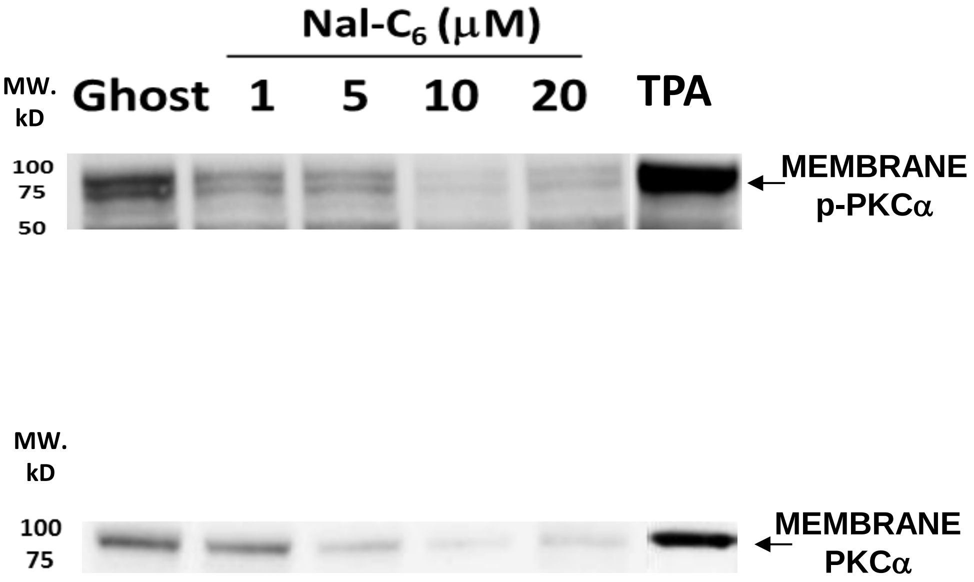

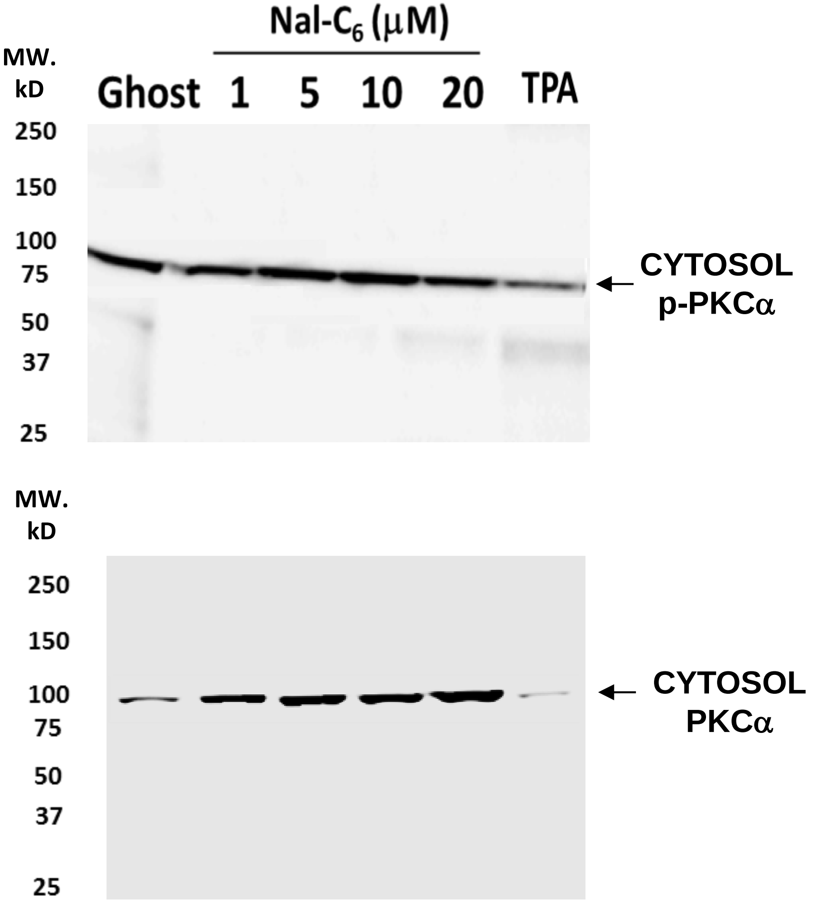

Figure 2 e

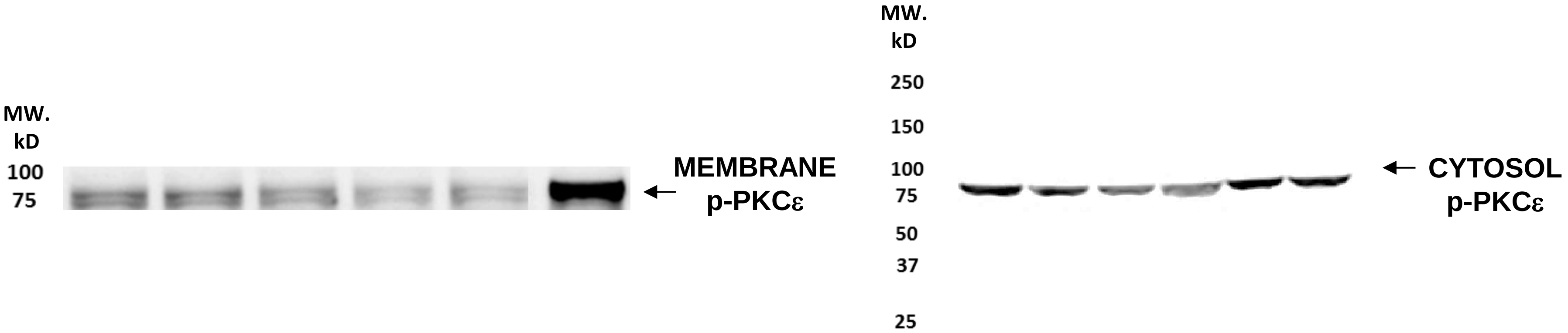

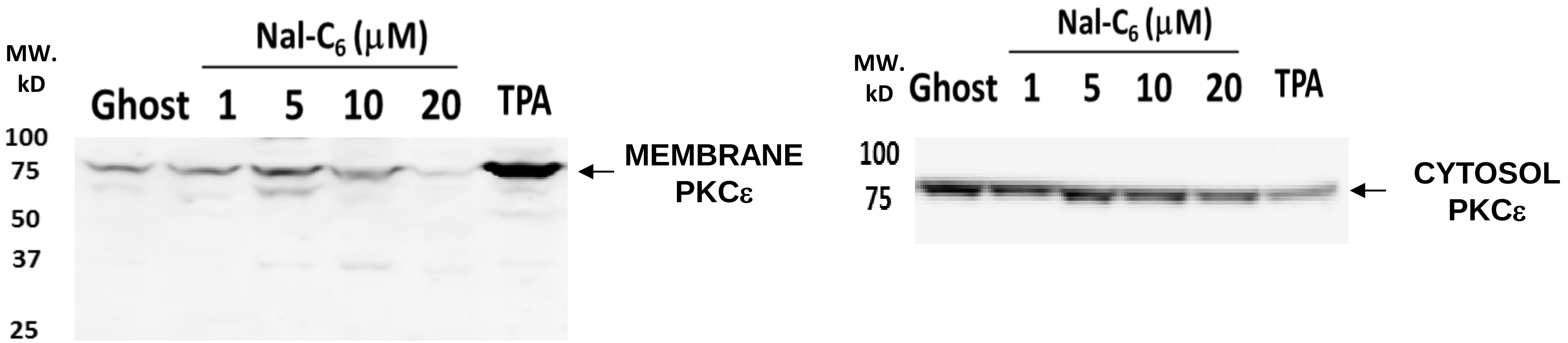

Figure 2 f

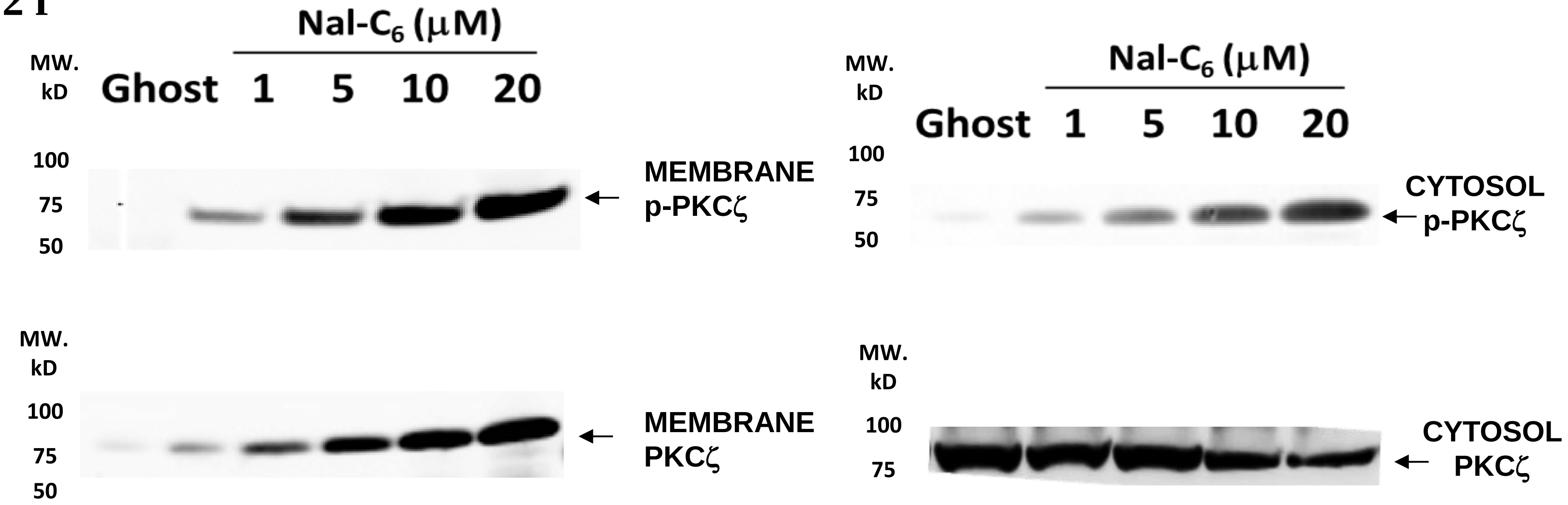

Figure 2 g

MDA-MB-231

Lu1205

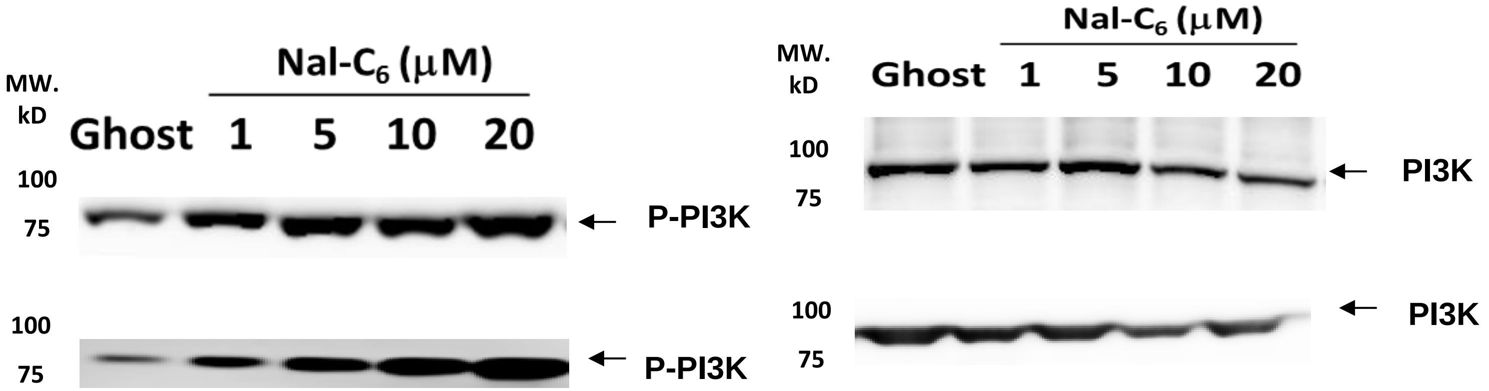

Figure 6 c

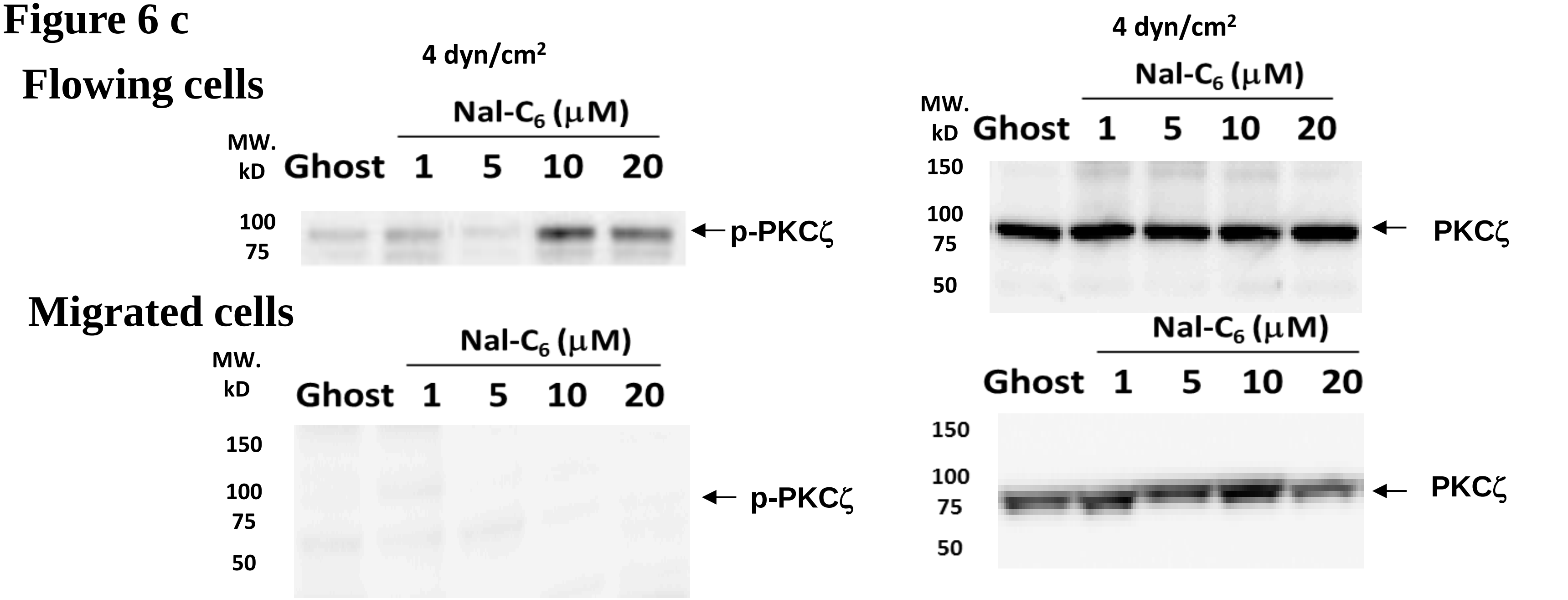

Figure 7 d

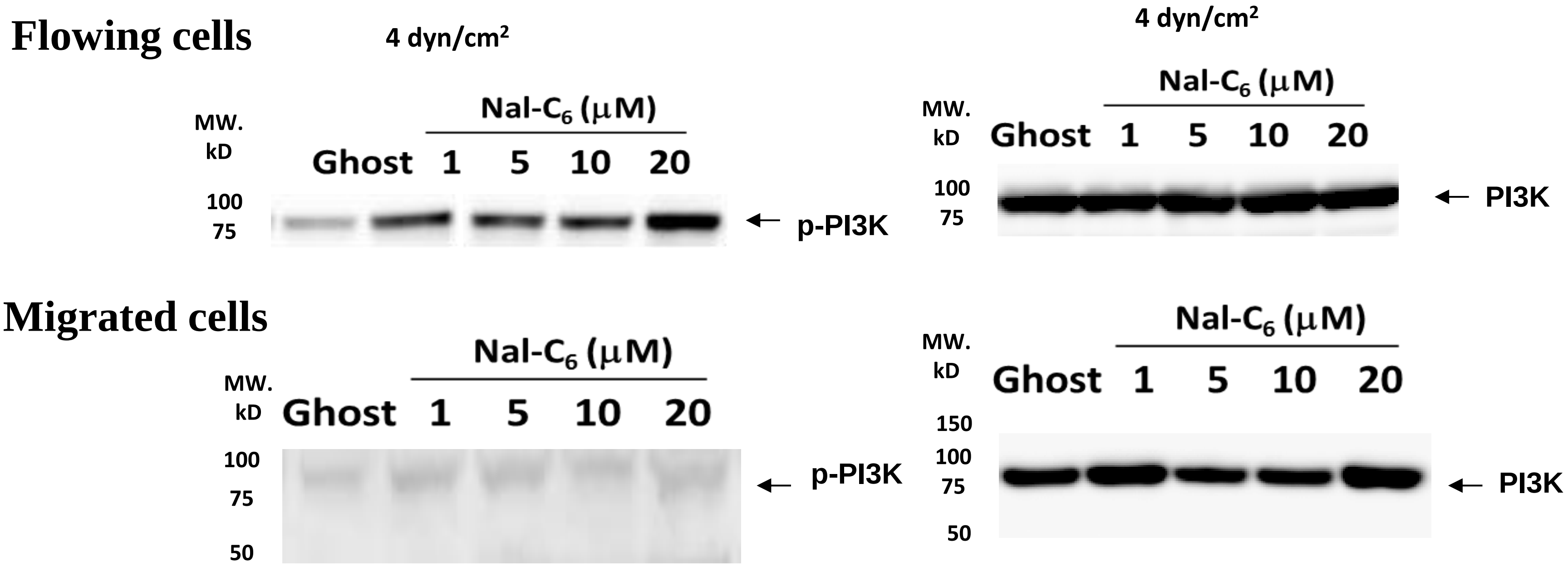

Figure 8 a

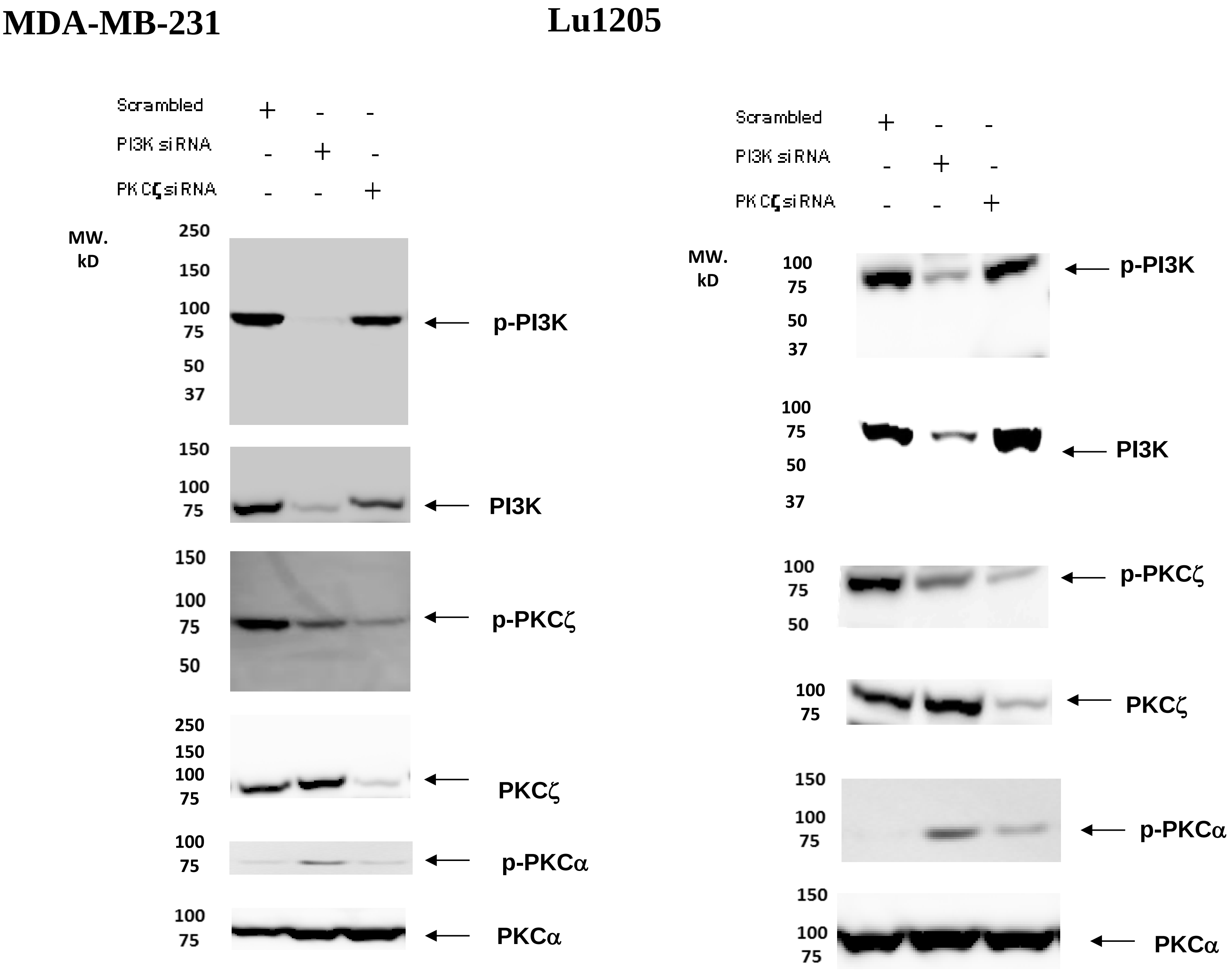
MDA-MB-231
Lu1205
Scrambled
PI3K siRNA
PKCζ siRNA
MW. kD
250
150
100
75
50
37
p-PI3K
PI3K
p-PKCζ
PKCζ
p-PKCα
PKCα

Figure 8 e

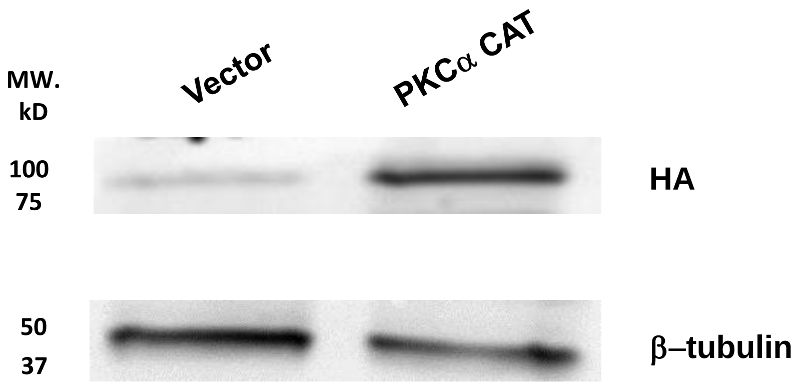

Supplement: Supplementary Information — Dataset 1 [file srep09275-s1.pdf]
